# Spatiotemporal translation of sperm acrosome associated proteins during early capacitation modulates sperm fertilizing ability

**DOI:** 10.1016/j.jare.2025.03.035

**Published:** 2025-03-18

**Authors:** Yoo-Jin Park, Won-Ki Pang, Do-Yeal Ryu, Md Saidur Rahman, Myung-Geol Pang

**Affiliations:** Department of Animal Science & Technology and BET Research Institute, Chung-Ang University, Anseong, Gyeonggi-do 17546, Republic of Korea

**Keywords:** Spermatozoa, Fertility, Translation, Capacitation, Translocation, Fluorescent noncanonical amino acid tagging system

## Abstract

**Introduction:**

Despite the lack of essential cytoplasmic organelles in mature spermatozoa, which creates unfavorable conditions for transcription and translation, the presence of various mRNA and proteins during capacitation suggests potential for de novo protein synthesis.

**Objectives:**

We applied a metabolic labeling method using a fluorescent noncanonical amino acid tagging system (FUNCAT) and proximity ligation method (PLA) in normal and reduced fertility spermatozoa to detect different translation phenomena during sperm capacitation according to their fertility.

**Methods:**

We explored different proteome changes in spermatozoa according to the time-sequential capacitation process (0, 20, 40, 60, and 120 min) between normal [average fertility rate (FR) = 77.44 % ± 1.51] and reduced fertility (average FR = 58.57 % ± 1.64) spermatozoa bull spermatozoa, as the representative male fertility models owing to their broad spectrum of fertility phenotypes. Moreover, the FUNCAT/PLA method was used to detect and visualize different translation phenomena during sperm capacitation according to fertility.

**Results:**

We found that sperm-associated protein (SPACA) 1 and SPACA5 were newly synthesized in the head of normal-fertility spermatozoa, whereas a lack of newly synthesized proteins in the head and a relatively earlier loss of SPACA1 and SPACA5 were observed in the reduced-fertility spermatozoa. Moreover, the mitochondrial translation inhibitor, chloramphenicol, partially inhibited sperm translation and delayed translocation, suggesting that mitochondria participate in sperm translation.

**Conclusion:**

Our results unveil time-sequential microenvironmental changes in sperm proteomes during capacitation, which lead to the orchestra of proteins that complete fertilization. Fertile spermatozoa are selected through inter-competition during the journey of fertilization in the female reproductive tract. This study provides an overview of how translation dynamics acts on the sperm selection and influence the evolution of sperm fertility.

## Highlights

• Spatiotemporal translation during sperm capacitation relates to fertility.
• Newly synthesized proteins were detected in spermatozoa by FUNCAT assay.
• Translation during the early capacitation introduces distinct male fertility.
• SPACAs are actively synthesized at early capacitation time.
• Inhibition of mitochondrial translation delayed translation of SPACAs.

## Graphical abstract

**Figure:**
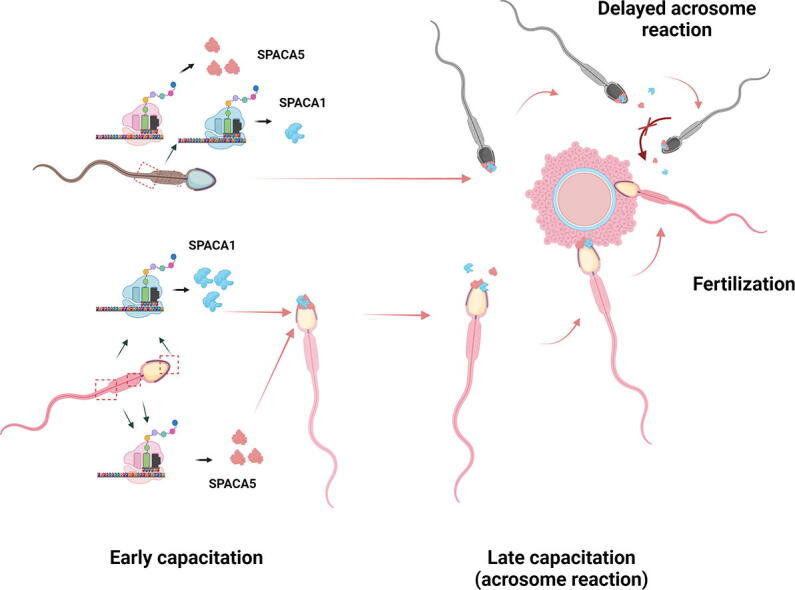

## Introduction

Infertility and unintended pregnancies are the major causes of family planning problems worldwide. Most contraceptive methods concentrate on women, but contraceptive methods for men are limited, even though half of fertility outcomes are derived from men [1]. Some efforts using hormones and non-hormonal small molecules to interfere with sperm cell development and maturation provide high accuracy in preventing pregnancy but are time-consuming and lead to inescapable side effects [2]. Therefore, new strategies involving safe and effective molecules are necessary to improve male contraceptives.

Spatiotemporal processes involved in the formation of the proteome from genomic information, including transcription, mRNA translation, and protein degradation, maintain homeostasis and plasticity in cells [3], [4]. Several intracellular organelles, including ribosomes, the endoplasmic reticulum, and the Golgi apparatus, are necessary for coordinating compartmentalized biogenesis. Uniquely, mature spermatozoa, known as streamlined cells, are not encumbered by these essential organelles, which allows them to exhibit high motility and efficiently deliver paternal genetic information to oocytes. Moreover, sperm chromatin is tightly condensed because histones are replaced by protamine during the later stages of spermiogenesis [5]. Their distinct structural specialization reflects the functional changes in the sperm that occur in the absence of either transcription or translation after leaving the testis and from epididymal maturation to fertilization [6], [7], [8]. Therefore, serial changes by spermatozoa to acquire fertilizing ability during maturation or capacitation, including increased flagella beating, cholesterol efflux, chemotaxis, intracellular calcium levels, and membrane fluidity, have been considered the result of post-translational modifications [9], [10]. However, notwithstanding the unfavorable conditions involved in the transcription and translation of spermatozoa, some evidence, such as the presence of varied mRNA in mature spermatozoa [11], [12], [13] and the increased relative abundance of several proteins during sperm maturation and capacitation [7], [14], [15], [16], has raised the possibility of translation in spermatozoa.

Proteostasis is regulated by a multicompartmental system, including protein synthesis, folding, trafficking, and clearance, to maintain cellular function and viability [17]. In the mitochondria, both nuclear and mitochondrial genomes participate in mitochondrial proteostasis to produce and construct a functional respiratory chain, the oxidative phosphorylation (OXPHOS) complex [18]. It is commonly believed that mitochondrial translation by mitochondrial ribosomes (mitoribosomes) is associated with inner mitochondrial membrane proteins consisting of 13 core OXPHOS subunits encoded on mitochondrial DNA that are co-assembled with nuclear DNA-encoded OXPHOS subunits [18], [19]. However, recent studies have reported the functional connection between cytosolic and mitochondrial ribosomes for the regulation of protein homeostasis [20], [21], [22]. Mitochondrial ribosomal protein 18 (MRPL18), present in the cytosol, accelerates heat-shock protein translation through its incorporation into 80S ribosomes and permits the engagement of heat-shock protein mRNAs following exposure to stress [22]. Moreover, the possibility of nuclear-encoded mRNA translation by mitoribosomes during sperm capacitation is being considered [16].

**Fig. 1:**
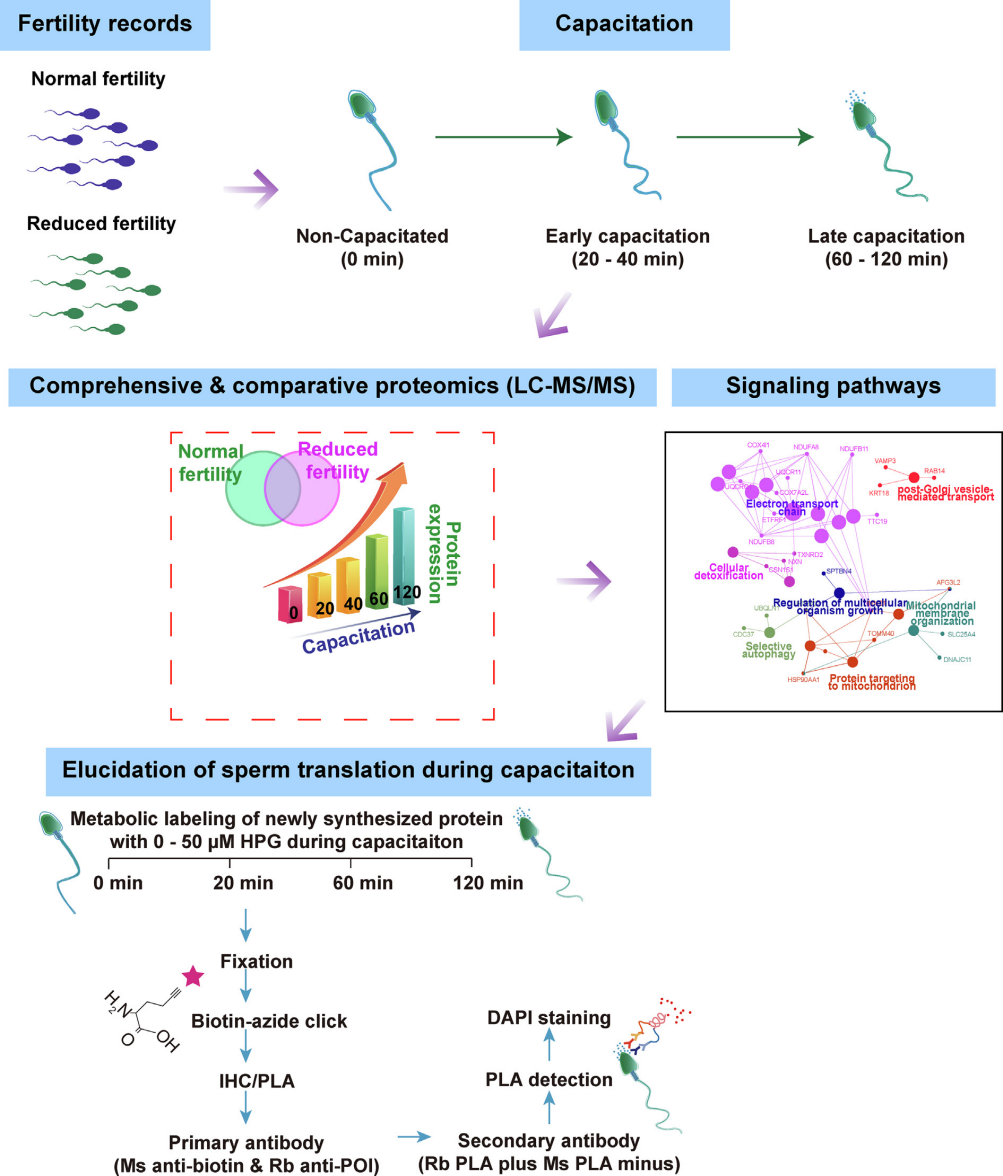
**Strategies to address the spatiotemporal translation during sperm capacitation using combined methods with proteomic and FUNCAT/PLA.** Quantitative LC-MS/MS was performed to identify different time-sequential changes in the bovine sperm proteomes between normal and reduced fertility during sperm capacitation. Fertility-dependent proteins upregulated during sperm capacitation were used to analyze the signaling pathways. The FUNCAT–PLA protocol was used to label newly synthesized proteins and specific proteins of interest (POI) in spermatozoa during capacitation. During sperm capacitation, HPG, biotinylated using click chemistry, was incorporated into the newly synthesized proteins. An in situ proximity ligation assay (PLA) was performed using antibodies for HPG-tagged proteins (orange Y) and POI (blue Y) to detect proximity, which was fluorescently labeled red by signal amplification. (For interpretation of the references to colour in this figure legend, the reader is referred to the web version of this article.)

Despite recent publications on sperm translation, we lack insights regarding the precise mechanisms involved in the translation of proteins in spermatozoa from capacitation to fertilization and their contribution to fertilization. Therefore, we expect that the studies on the molecular basis associated with time-sequential changes in spermatozoa from capacitation to fertilization will improve our understanding of male fertility complexity. First, different time-sequential changes in proteomes during capacitation, depending on fertility, were identified by quantitative mass spectrometry to recapitulate physiologically relevant changes in spermatozoa. Interestingly, we found differences in the upregulated or newly detected proteins between normal and reduced fertility spermatozoa following capacitation. Therefore, we investigated whether the proteins upregulated during sperm capacitation were derived from sperm translation by visualizing the spatial coincidence of newly synthesized proteins during sperm capacitation according to sperm fertility using a fluorescent non-canonical amino acid tagging system (FUNCAT) (Fig. 1).

## Materials and methods

### Semen source, classification of fertility, and induction of capacitation

All animal experiments were approved by the Institutional Animal Care and Use Committee of Chung-Ang University, Seoul, Korea (IACUC Number: 2016 − 00009). In this study, bovine spermatozoa were considered an ideal model for male fertility, that is, normal and sub-fertility, owing to the availability of ample amounts of homogenous semen samples, with a broad fertility range [23]. Ten representative semen samples were procured from Hanwoo bulls (Korean native cattle) with more than three years of fertility history and at least 100 years of total breeding. Frozen-thawed bovine semen samples from bulls with normal and reduced fertility were centrifuged for 20 min at 400 × g with a discontinuous Percoll density gradient, consisting of 1 mL of 90 % Percoll and 1 mL of 45 % Percoll, to remove extender debris and dead spermatozoa. To mimic the consecutive changes that the spermatozoa undergo while moving through the female reproductive tract, the spermatozoa were incubated in Tyrode’s albumin lactated pyruvate (TALP) medium supplemented with 10 μg/mL heparin (capacitation medium, CM) (Sigma-Aldrich, St Louis, MO, USA) in vitro, as described in our previous study, which helped optimize the capacitation conditions of the bull spermatozoa [24]. The TALP medium consisted of 100 mM NaCl, 3.1 mM KCl, 2.0 mM CaCl·2H_2_O, 0.4 mM MgCl·6H_2_O, 0.3 mM Na_2_HPO_4_·12H_2_O, 21.6 mM sodium lactate, 25 mM NaHCO_3_, 1.0 mM sodium pyruvate, and 0.6 % bovine serum albumin. Spermatozoa from bulls with normal and reduced fertility were functionally capacitated and collected at five time points (0, 20, 40, 60, and 120 min). To inhibit the mitochondrial translation during sperm capacitation, spermatozoa were incubated with a mitochondrial translation inhibitor [0.1 mg/mL of D-chloramphenicol (CP)] [16] during sperm capacitation.

### Sperm preparation for proteomic analysis

Spermatozoa (1 × 10^6^ sperm cells) were lysed in sodium dodecyl sulfate (SDS) sample buffer consisting of 65.8 mM Tris–HCl, 1 % SDS, 10 % glycerol, 0.01 % bromophenol blue, and 5 % β-mercaptoethanol for 1 h at room temperature, and each sample was loaded into a 12 % SDS–polyacrylamide gel (SDS–PAGE). The electrophoresed proteins were visualized using Coomassie Brilliant Blue, and each lane was excised for liquid chromatography tandem mass spectroscopy (LC–MS/MS) analysis. Following de-staining with de-staining buffer (10 mM ammonium bicarbonate and 50 % acetonitrile (ACN)), the gels were dried, reduced using 10 mM dithiothreitol, and alkylated with iodoacetamide. Tryptic digestion was performed using 50 mM ammonium bicarbonate, 50 % acetonitrile, and 5 % trifluoroacetic acid (TFA), and the digested peptides were lyophilized. For LC–MS/MS analysis, the lyophilized peptides were resuspended in 0.5 % TFA.

### Proteomic analysis using LC–MS/MS

Tryptic peptide samples were loaded into an Ultimate 3000 UPLC system (Dionex, Sunnyvale, CA, USA), connected to Q Exactive Plus mass spectrometer (Thermo Scientific, Waltham, MA, USA), and equipped with nanoviper trap column (15 cm x 75 µm, Thermo Scientific) and nanoviper analysis column (100 µm x 2 cm; Thermo Scientific) at a flow rate of 300 nL/min. Peptides were eluted by a gradient of 5 – 40 % ACN for 95 min. All MS and MS/MS spectra captured by the Q Exactive Plus mass spectrometer were obtained in the data-dependent top 12 mode, with full scans of MS/MS acquisition at a resolution of 120,000 (*m*/*z* 400–1600) in the Orbitrap. MS/MS data were searched with MASCOT 2.7 (Matrix Science Inc., Boston, MA, USA), using parameters corresponding to a false discovery rate (FDR) < 1 %. MS/MS analysis was performed at least three times for each sample and in-gel digestion and LC–MS/MS analysis was conducted at the Korea Basic Science Institute (Ochang Headquarters, Division of Bioconvergence Analysis) [25].

### Signaling pathway analysis

Pathway enrichment analysis of upregulated and newly synthesized proteins in spermatozoa during early and late capacitation was conducted using g:Profiler and visualized by ClueGo Cytoscape application (Cytoscape Version 3.8.2). StringApp Cytoscape application was used to identify cellular components GO terms of interest proteins and visualize the protein–protein interaction network (FDR < 0.05).

### Western blot analysis

Sperm cell lysates were loaded onto a 12 % SDS-PAGE, electrophoresed, and transferred onto polyvinylidene difluoride membranes (Amersham). Next, the membranes were blocked in 5 % skim milk for 1 h and incubated with anti-SPACA1 (Abcam), anti-GAPDH (Abcam), and GPx4 (LifeSpan Biosciences) antibodies overnight at 4 ℃. The membranes were then washed with PBS containing Tween 20 and incubated with the specified peroxidase-conjugated secondary antibody at room temperature for 2 h. The proteins were detected using a Chemiluminescence Western Detection Kit (Amersham).

### Metabolic labelling with HPG and biotin-azide click (FUNCAT)

Metabolic labeling of the newly synthesized proteins was performed using the Click-iT L-homopropargylglycine (HPG) cell reaction kit (Invitrogen, Carlsbad, CA, USA) during sperm capacitation, according to the manufacturer’s recommendations and the following modifications: Percoll-separated spermatozoa were resuspended in CM supplemented with 50 µM HPG and centrifuged at 400*g* for 5 min. Spermatozoa were then incubated for 20–120 min to allow for metabolic labeling during capacitation. Metabolically labeled spermatozoa were washed twice with PBS at 400*g* for 5 min and fixed in 4 % paraformaldehyde for 20 min at room temperature. The spermatozoa were washed twice for 5 min with PBS, smeared on glass slides, and air-dried. Then, the spermatozoa were permeabilized with PBS containing 0.05 % Triton-X-100 for 20 min, washed 3 × three times with PBS for 5 min each, and blocked with 3 % BSA for 30 min. Metabolic-labeling of proteins was performed using the copper-catalyzed (3 + 2)-azide-alkyne-cycloaddition chemistry (CuAAC) click reaction, as described previously [26] with some modifications. A click-reaction cocktail comprised a 1 × click-reaction buffer, a 25 µM biotin-azide tag (PEG4 carboxamide-6-azidohexanyl biotin), 2 mM CuSO4, a 1 × click-iT cell buffer additive, and 5 mM click-iT cell buffer additive. A biotin monoclonal antibody (Invitrogen) was used to detect HPG-biotin-azide-tagged proteins, and the samples were subsequently stained with a secondary antibody coupled to Alexa Fluor 488 (Invitrogen, Eugene, OR, USA). The spermatozoa were counterstained and mounted using Vectashield (Vector Laboratories, Burlingame, CA, USA). Images were acquired using a confocal microscope (LSM 800, Carl Zeiss, Germany) and analyzed using ZEN v2.6.

### Proximity ligation assay (PLA)

The close proximity of the newly synthesized proteins and SPACA1 proteins was detected using an anti-biotin antibody in combination with a SPACA1-specific antibody (Abcam) and visualized using Duolink reagents (Sigma), according to the manufacturer’s recommendations. Briefly, spermatozoa metabolically labeled with HPG and biotin-azide click reaction buffer were blocked in PBS containing 3 % BSA (blocking buffer) for 30 min, incubated with primary antibody pairs, and diluted in blocking buffer for 1 h. Following the incubation with rabbit PLAplus and mouse PLAminus probes as secondary antibodies for 1 h at 37 ℃, the spermatozoa were washed twice for 5 min with wash buffer A before a ligation reaction was performed using the circularization oligos and T4 ligase (Duolink Detection reagents Red, Sigma) in a prewarmed, humidified incubator at 37 ℃ for 30 min. After washing with wash buffer A, amplification was conducted using 1 × amplification reaction mixture (Duolink Detection reagents Red, Sigma) in a prewarmed, humidified incubator at 37 ℃ for 2 h. Finally, the spermatozoa were washed twice for 10 min with 1 × wash buffer B and 1 min with 0.01 × wash buffer B and mounted using Vectashield (Vector Laboratories).

### Field emission transmission electron microscope (FE-TEM)

HPG-tagged spermatozoa were fixed in 2.5 % glutaraldehyde solution (Sigma-Aldrich), post-fixed in 1 % osmium tetroxide, and stained with 0.5 % uranyl acetate. Following dehydration in a graded ethanol series, the spermatozoa were embedded and sectioned at 5 µm. The sections were stained with 2 % uranyl acetate in methanol for 20 min, followed by lead citrate for 5 min, and then analyzed by FE-TEM (JEM-F200, Jeol, Tokyo, Japan) using an acceleration voltage of 80 kV.

### Statistical analysis

Proteomic data were normalized and analyzed using MaxQuant and Perseus (version 1.6.2). Principal component analysis (PCA) based on Bray–Curtis dissimilarity was performed using R software (RStudio, Boston, MA, USA). A comparison of protein expression and cell population during capacitation between the NF and RF groups was carried out using two-way ANOVA in GraphPad Prism (Version 10.1.1, San Diego, California, USA) with Šídák's multiple comparison test. The Chi-square test was used to determine the differences in the percentages of newly synthesized and upregulated proteins during early and late capacitation between the normal and reduced fertility groups.

## Results

### Different sperm proteomes between normal and reduced fertility at different capacitation time

**Fig. 2:**
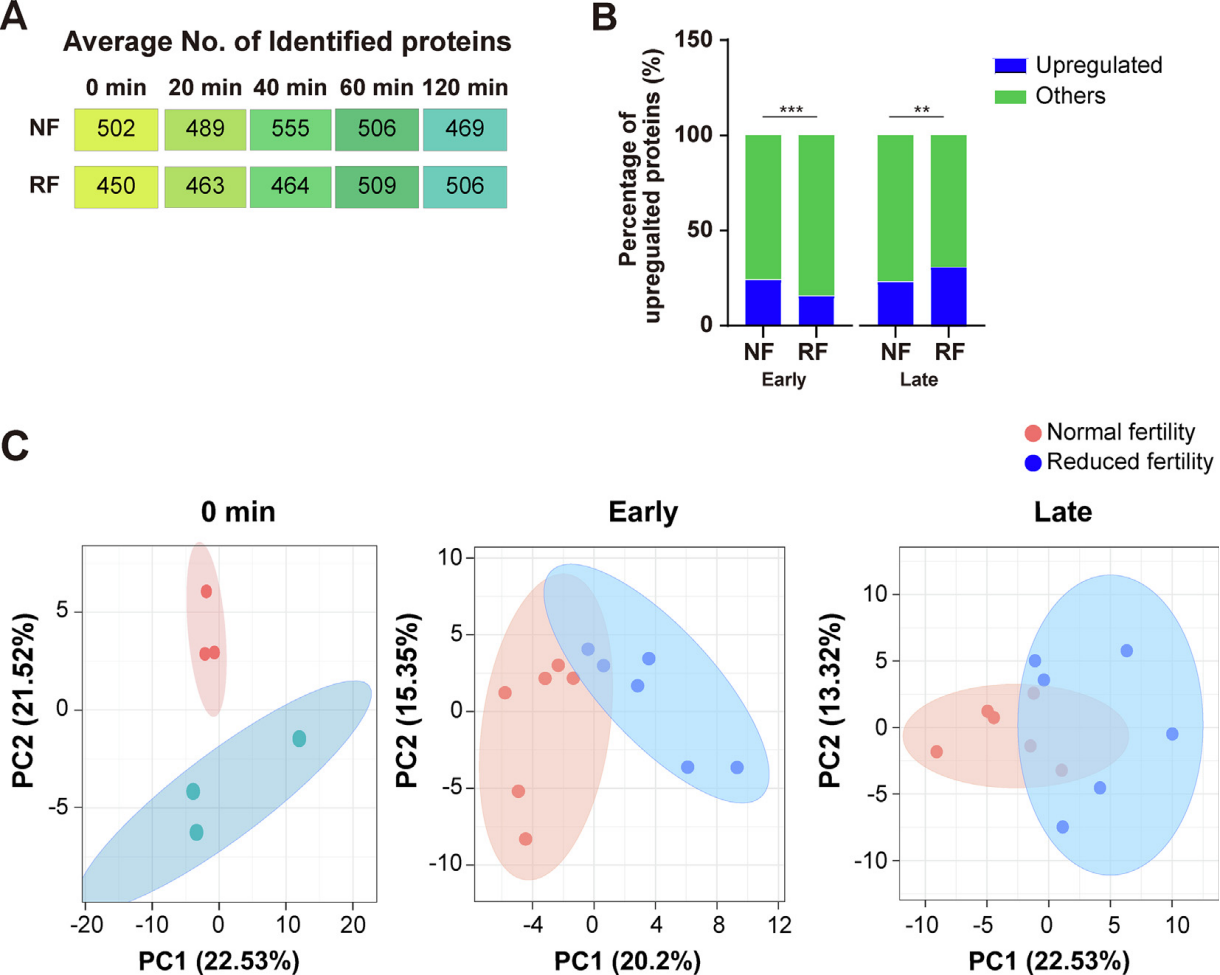

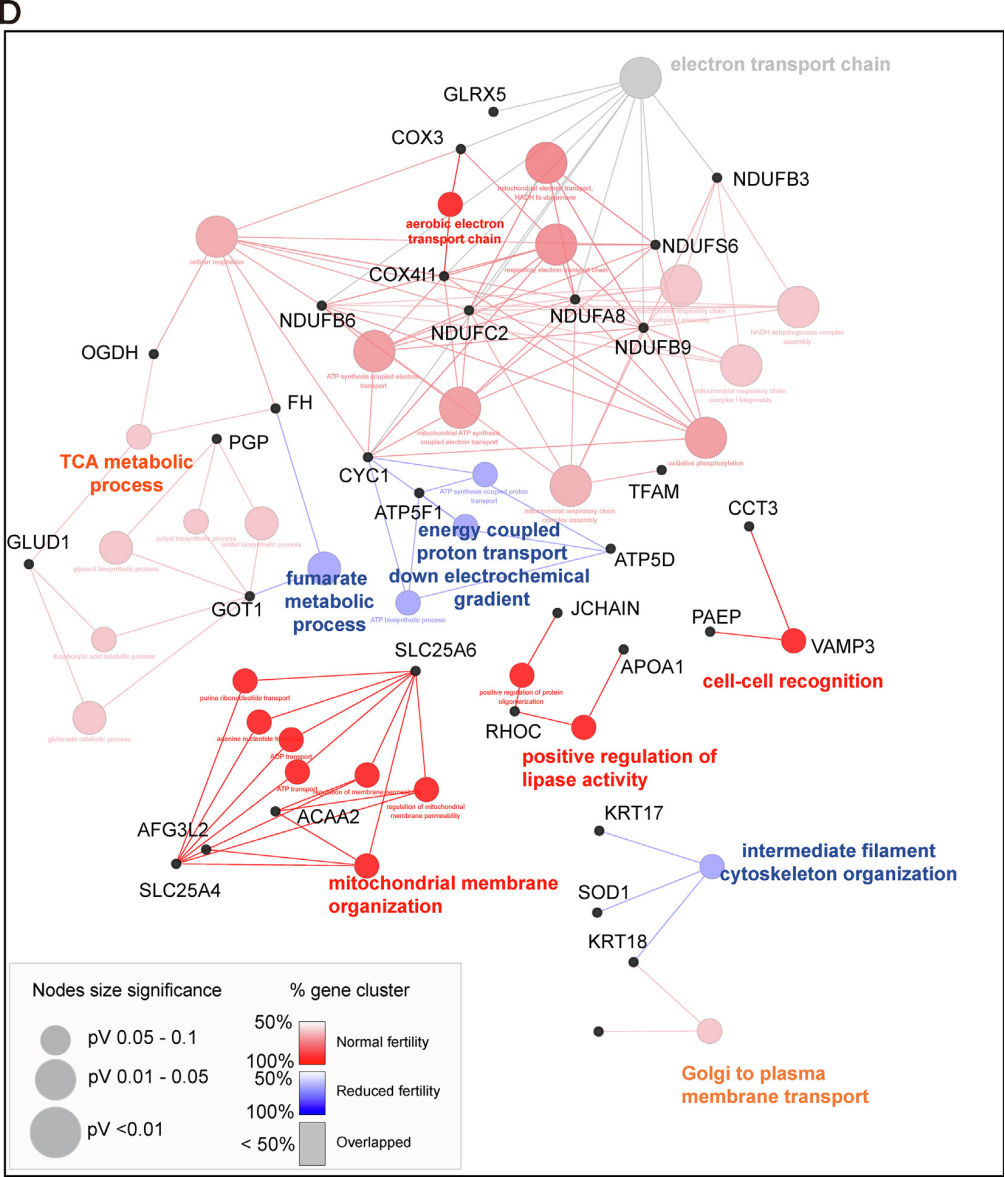

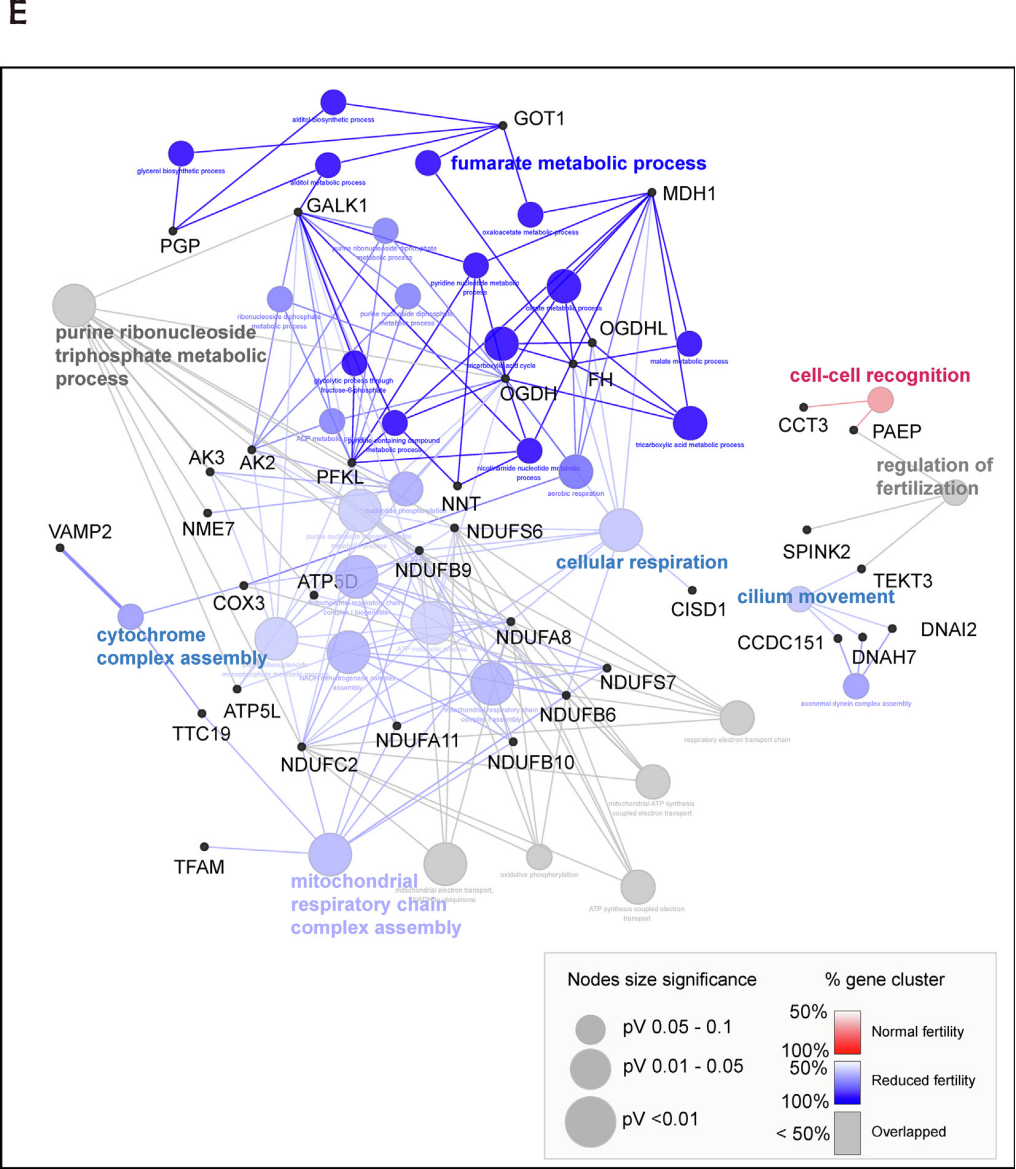

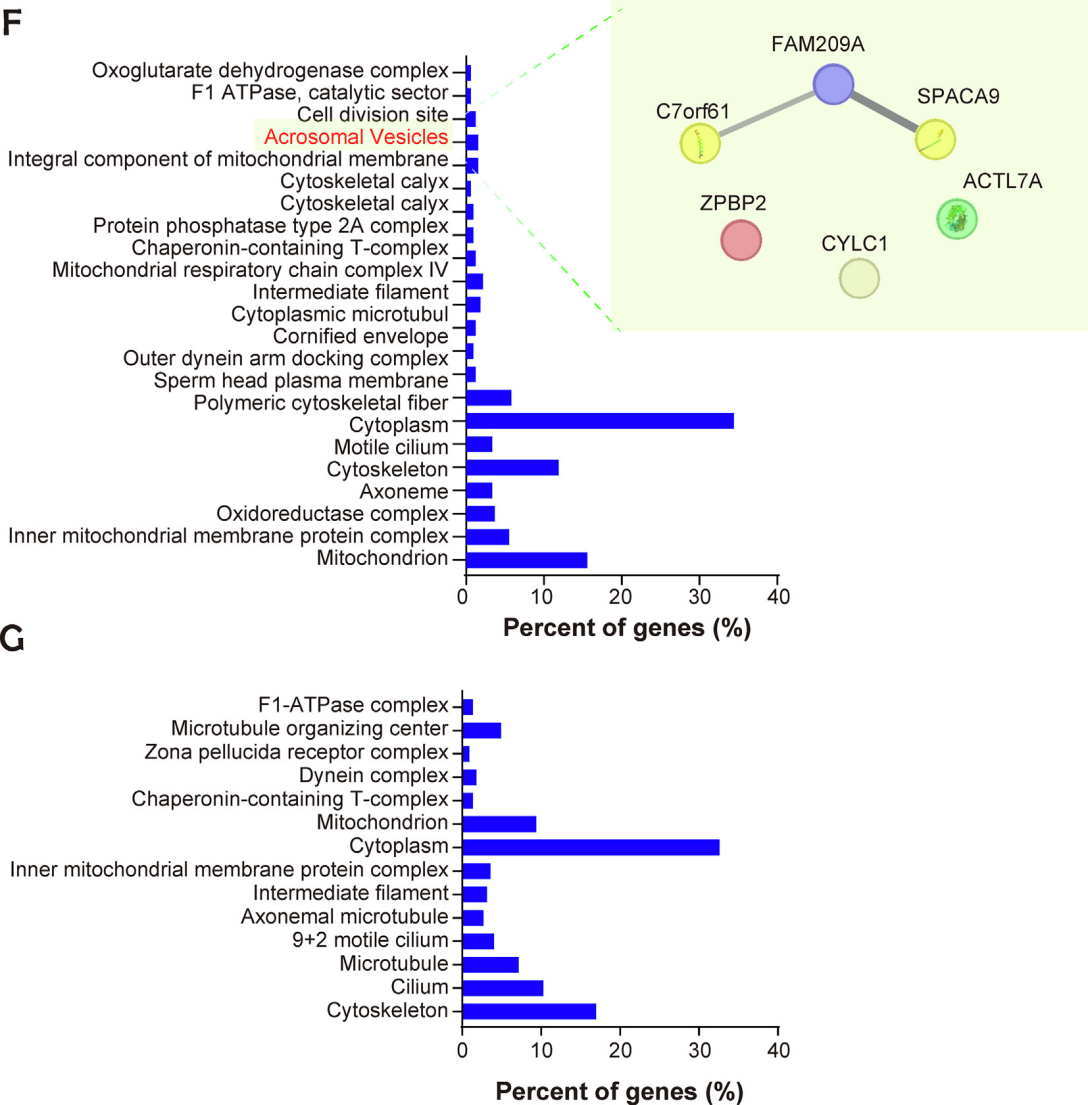
**Substantial changes of sperm proteomes during capacitation according to fertility.** (A) Average number of proteins identified within each population. (B) Percentage of upregulated and newly synthesized proteins during early and late capacitation between normal and reduced fertility spermatozoa. The Chi-square test was used to evaluate the differences between normal and reduced fertility spermatozoa. ^**^P < 0.01, ^***^P < 0.001. (C) Principal component analysis (PCA) of the proteome for each sperm cell population with their respective biological replicates. Each plot shows the capacitation status of spermatozoa (0 min: non-capacitated; 20–40 min: early capacitation; 60–120 min: late capacitation), and the colors of the symbols denote the fertility status of the spermatozoa (normal fertility = red; reduced fertility = blue). (D) ClueGo-enriched pathways of upregulated and newly detected proteins in normal (red) and reduced fertility (blue) spermatozoa during early capacitation. (E) ClueGo-enriched pathways of upregulated and newly detected proteins in normal (red) and reduced fertility (blue) spermatozoa during late capacitation. (F and G) Gene Ontology analysis of cellular components in upregulated proteins during early capacitation of normal (F) and reduced (G) fertility spermatozoa. String App Cytoscape application was used to analyze signaling pathways and enrichment (FDR < 0.05). (For interpretation of the references to colour in this figure legend, the reader is referred to the web version of this article.)

Analysis of the capacitated status using chlortetracycline staining showed that the capacitated spermatozoa were enriched in the normal and reduced fertility groups at capacitation times of 20 min and 40 min, which were categorized as “early capacitation,” while the subset of the sperm population that underwent the acrosome-reaction, which is a concomitant process of capacitation that allows the spermatozoa to penetrate and fuse with oocytes [27], was detected at 60 min and 120 min and classified as “late capacitation” (Supplementary Fig. S1B). Sperm motility decreased in a time-dependent manner, while kinematic motion parameters, including straight-line velocity, average path velocity, linearity, and mean amplitude of head lateral displacement, increased during sperm capacitation, regardless of fertility (Supplementary Table S1). Before capacitation, 502 and 450 proteins were identified in the spermatozoa with normal and reduced fertility, respectively. Following time-sequential sperm capacitation under normal fertility conditions, 489, 555, 506, and 469 proteins were identified at 20, 40, 60, and 120 min, respectively. In reduced fertility conditions, 463, 464, 509, and 506 proteins were identified after 20, 40, 60, and 120 min of capacitation, respectively (Fig. 2A). Pearson’s correlation coefficient between biological replicates was higher than 0.96, indicating that the proteomic analysis was highly reproducible (Supplementary Fig. S2). Moreover, the percentage of upregulated and newly synthesized proteins in spermatozoa with normal fertility was significantly higher than that in spermatozoa with reduced fertility during early capacitation (Fig. 2B, P < 0.001). In contrast, the percentage of upregulated and newly synthesized proteins in reduced fertility spermatozoa was significantly higher than that in normal fertility spermatozoa during late capacitation (Fig. 2B, P < 0.01).

We explored the changes in the proteomes of normal and reduced fertility spermatozoa according to the time-sequential capacitation process. In this study, bovine spermatozoa were used as representative male fertility models because of their broad spectrum of fertility phenotypes and biological and genetic similarities with other mammals The fertility rate (FR), defined as the ratio of the number of cows that did not show a gain in estrus up to 60 days post-insemination to the total number of inseminated cows, was categorized into normal (n = 5, average FR = 77.44 % ± 1.51) and reduced fertility (n = 5, average FR = 58.57 % ± 1.64) (Supplementary Fig. S1A, P < 0.01). To investigate the differences in sperm proteomes during capacitation and according to fertility, we collected functionally capacitated spermatozoa for 0, 20, 40, 60, and 120 min from bulls with normal and reduced fertility for analysis using LC-MS/MS with a nano-electrospray ion source.

### Different time-sequential changes in sperm proteomes during capacitation according to fertility

During early capacitation, 173 proteins were upregulated or newly detected in the normal fertility group, whereas 106 proteins were upregulated or newly detected in the reduced fertility group (Supplementary Table S2 and Supplementary Fig. S3A). Gene Ontology (GO) pathway analysis of biological processes indicated that the proteins upregulated or newly synthesized during early capacitation were closely associated with the electron transport chain, regardless of fertility (Fig. 2D). Moreover, energy metabolism-associated signaling pathways were significantly enriched in the upregulated and newly detected proteins during early sperm capacitation; particularly, “fumarate metabolic process,” "intermediate filament cytoskeleton organization," and “energy coupled proton transport” were significantly enriched in reduced fertility spermatozoa, and the “TCA process,” “aerobic electron transport chain,” “mitochondrial membrane organization,” and “positive regulation of lipase activity” signaling pathways were significantly enriched in normal fertility spermatozoa (Fig. 2D). During late capacitation, 158 proteins were upregulated or newly detected under normal fertility conditions, whereas 224 proteins were upregulated or newly detected under reduced fertility conditions (Supplementary Table S3 and Supplementary Fig. S3B). Specifically, mitochondria activation-associated signaling pathways, including “cytochrome complex assembly,” “mitochondrial respiratory chain complex assembly,” and “cellular respiration,” were identified as the most significant biological processes that mapped to spermatozoa with reduced fertility that underwent late capacitation (Fig. 2E). Importantly, the proteins significantly upregulated in the normal fertility spermatozoa at the early capacitation stage were closely related to the “Golgi to plasma membrane transport” and “cell–cell recognition” signaling pathways, while no changes were detected in the reduced fertility group (Fig. 2D). Similarly, cellular components associated with “acrosomal vesicles” were uniquely identified in the upregulated proteins from the normal fertility spermatozoa (Fig. 2F and 2G). In particular, six proteins, FAM209A, C7orf61, ZPBP2, CYLC1, ACTL7A, and SPACA9, were identified in the normal fertility spermatozoa as the most significant proteins mapped to the acrosomal vesicles (Fig. 2F).

Notably, principal component analysis (PCA) of the proteome profiles in non-capacitated and early capacitated spermatozoa showed clustering according to fertility at different capacitation time points, whereas the proteomes in the spermatozoa that underwent the acrosome-reaction from the normal and reduced fertility groups clustered closely (Fig. 2C).

### Optimization of labeling newly synthesized proteins in spermatozoa during capacitation

**Fig. 3:**
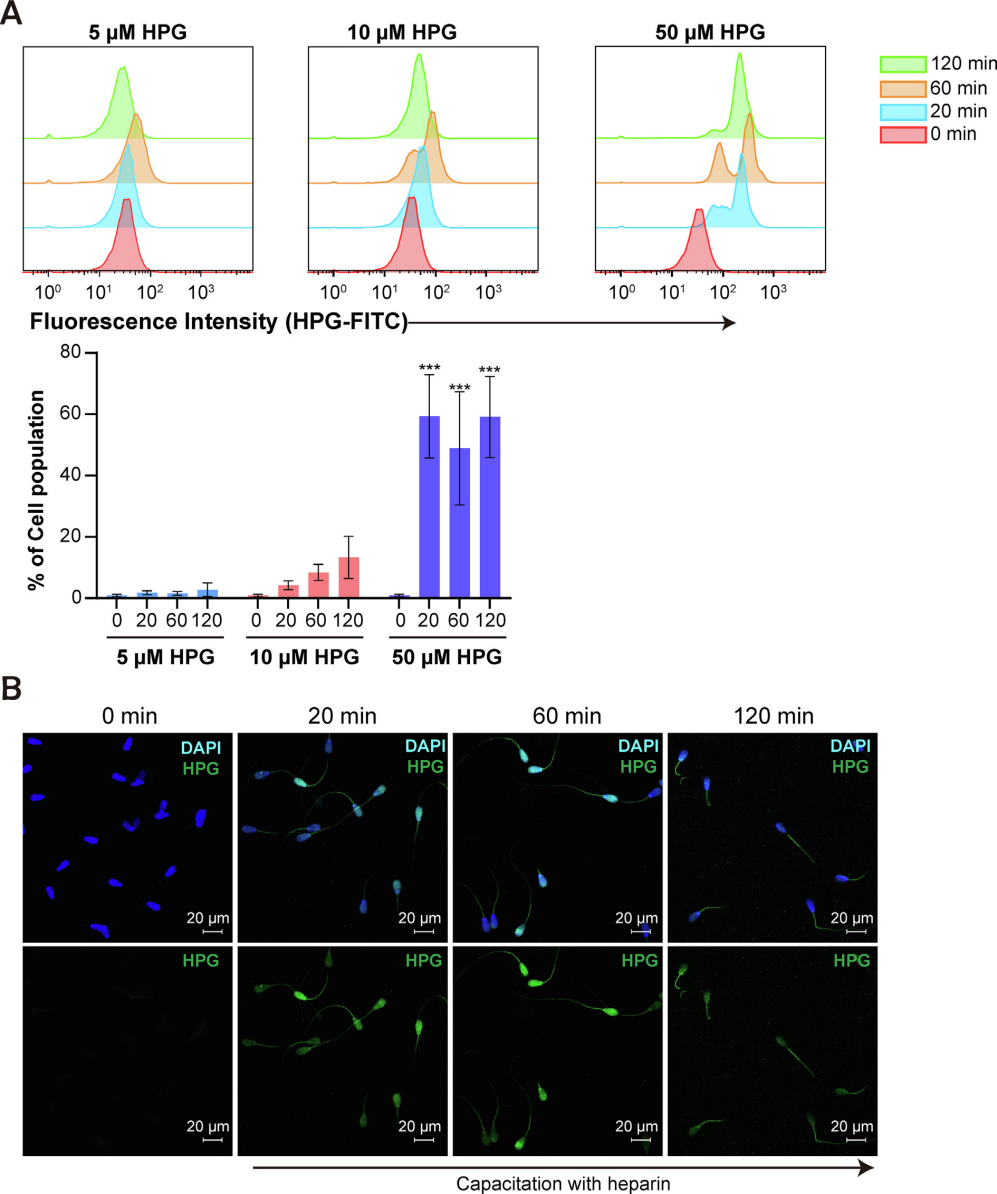

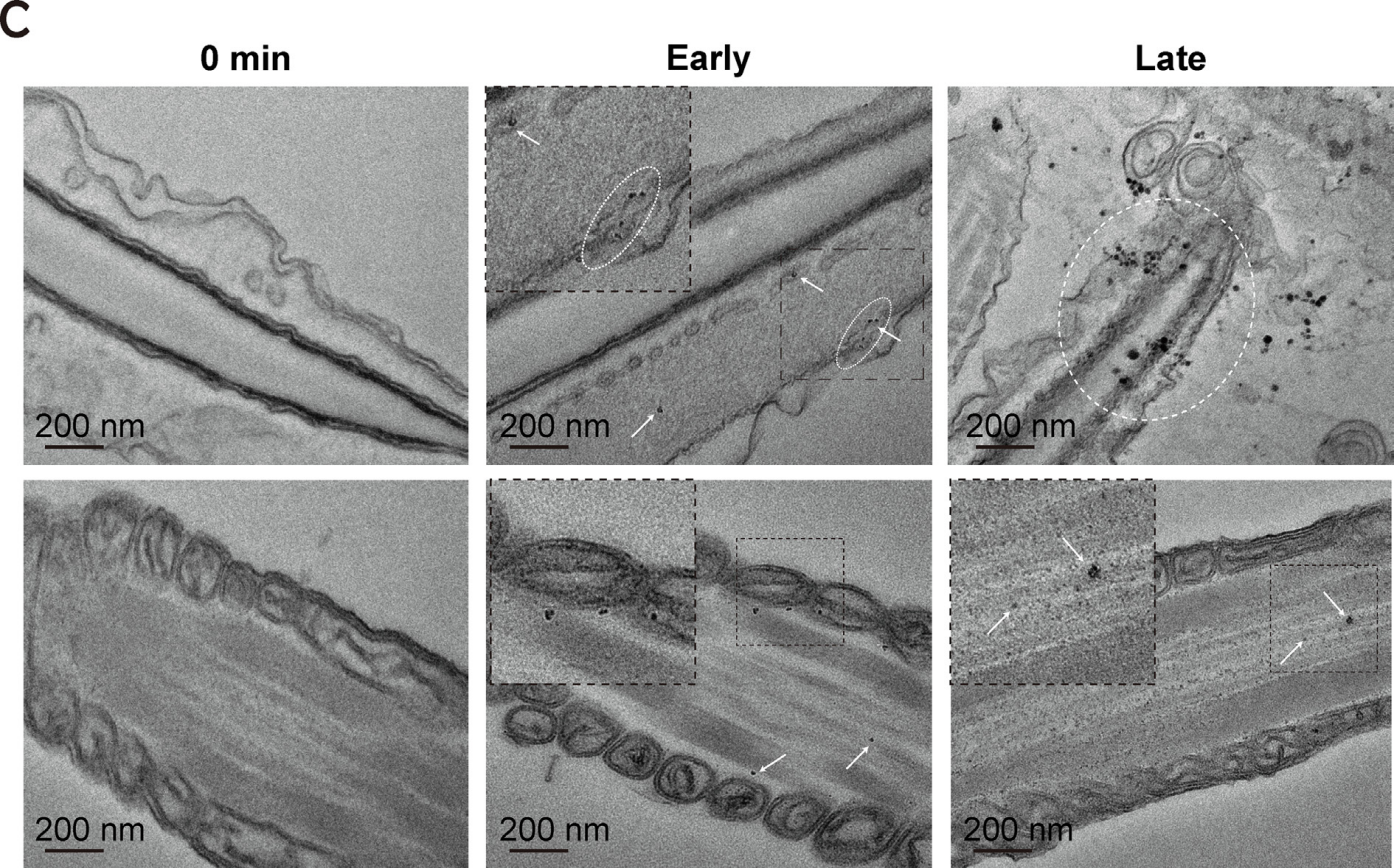
**Optimization of labeling newly synthesized proteins in spermatozoa with FUNCAT.** (A) Representative flow cytometry histogram of metabolically labeled spermatozoa. FUNCAT intensity in spermatozoa depending on incubation time (0, 20, 60, and 120 min) with different concentrations of HPG (0, 10, and 50 µM). Quantification of the incorporated proteins (left images) was enabled by a click reaction between alkyne (HPG) and Alexa Fluor® 488-azide. Statistical analysis of the dose-dependent accumulation percentage of HPG-labeled spermatozoa at different capacitation timepoints. Data represent the mean of three experiments ± SEM. ***P < 0.001 (n = 3, one-way ANOVA) vs. 0 min at 50 µM of HPG. (B) Representative immunofluorescence results after incorporation of 50 µM HPG into spermatozoa depending on capacitation time (0, 20, 60, and 120 min). (C) Localization of HPG-labeled proteins (white circle and black arrow) in spermatozoa was observed by transmission electron microscopy. The top-left inset shows the zoom of HPG incorporation into spermatozoa. Spermatozoa were incubated with 50 µM of HPG, depending on the incubation time (0, 20, and 120 min).

To investigate whether the proteins upregulated during sperm capacitation were derived from translation, the sperm were treated with an HPG and biotin-azide click system during sperm capacitation. To systematically optimize the HPG and biotin-azide click system in spermatozoa, various concentrations of HPG (0, 5, 10, and 50 µM) were added during sperm capacitation (0 to 120 min), and the HPG was tagged in situ with green fluorescently labeled probes (Fig. 1). The metabolic labeling efficiency was determined by flow cytometry, which revealed a linear relationship between the green fluorescence signal and HPG concentration (Fig. 3A). By comprehensively considering the high efficiency of HPG in detecting the newly synthesized proteomes in all capacitation conditions, an optimal HPG concentration of 50 µM was determined (Fig. 3A, P < 0.001). Fluorescently labeled newly synthesized proteins were detected in both the head and tail during capacitation (Fig. 3B). After capacitation for 120 min, weak signals for newly synthesized proteins were detected in the sperm head, whereas strong signals were observed in the mitochondrial region (Fig. 3B). Similarly, transmission electron microscopy (TEM) was used to analyze the metabolically labeled proteins that were detected in the heads and tails of spermatozoa during early capacitation, while coagulated protein particles were detected in the acrosomal membrane at late capacitation (Fig. 3C). HPG-labeled proteins were not detected in fully acrosome-reacted spermatozoa after 120 min of incubation (Supplementary Fig. S4).

### Inhibition of mitochondrial translation delays sperm capacitation

**Fig. 4:**
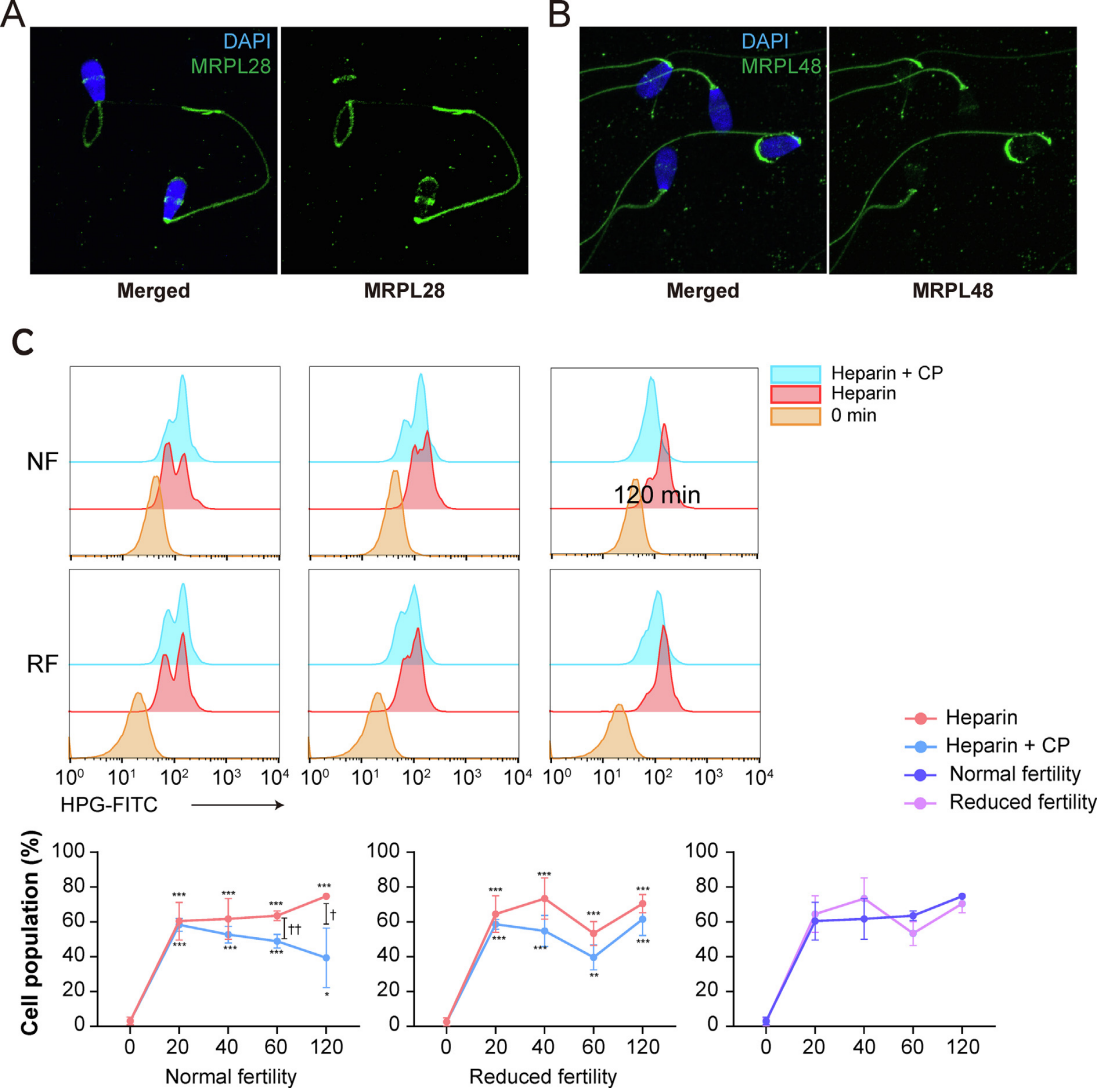

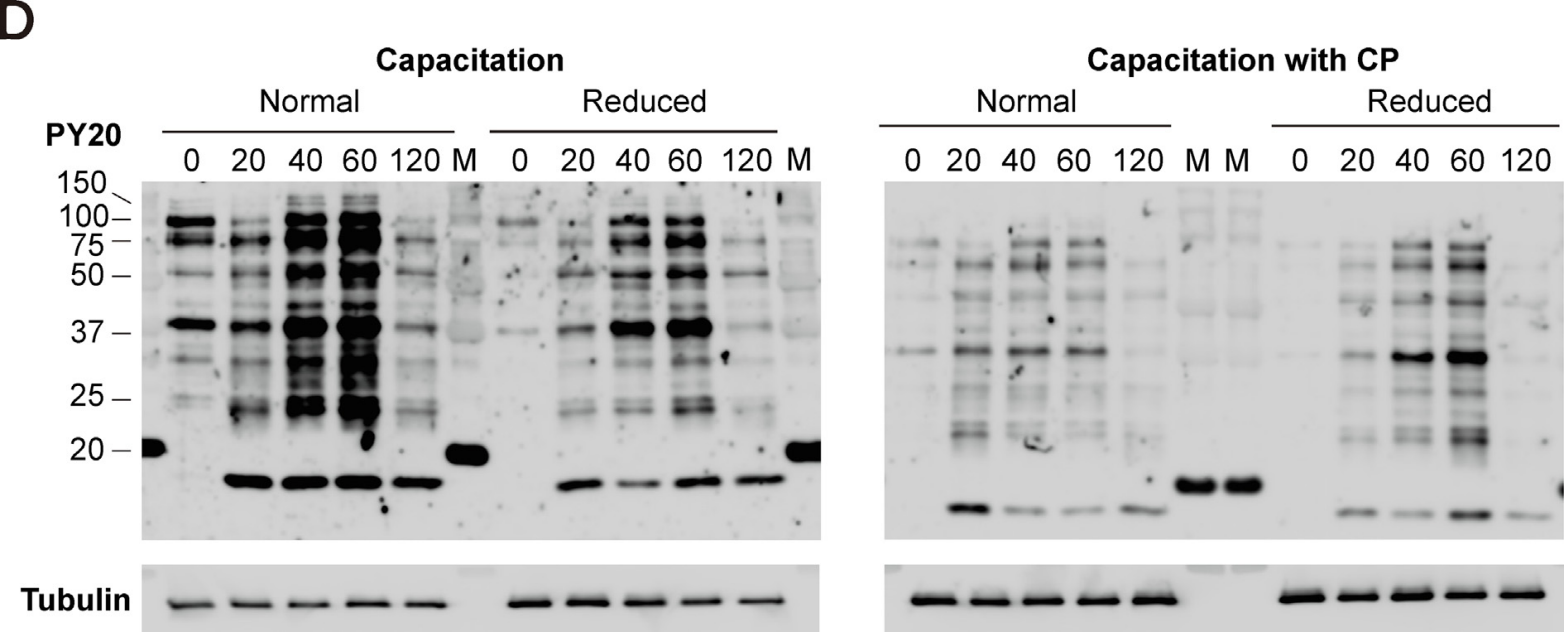
**Evidence of protein de novo synthesis during sperm capacitation.** Representative immunofluorescence images of spermatozoa after staining for mitochondrial ribosomal proteins (A) MRPL28 and (B) MRPL48 with Alexa 488 (green); the nuclei were stained with DAPI (blue). (C) Representative flow cytometry histogram of HPG-incorporated spermatozoa from normal and reduced fertility bulls in the presence or absence of the translation inhibitor CP following capacitation for 0, 20, 60, or 120 min. The percentage of HPG-incorporated spermatozoa with or without CP during sperm capacitation was determined by FACS analysis. Data represent the mean of three experiments ± SEM. *P < 0.05, **P < 0.01, ***P < 0.001 (n = 3) two-way ANOVA vs. 0 min. † < 0.05, †† < 0.01 (n = 3) two-way ANOVA; heparin vs. CP. (D) Western blot detection of phospho-tyrosine proteins in normal and reduced fertility spermatozoa during capacitation, with or without CP. (For interpretation of the references to colour in this figure legend, the reader is referred to the web version of this article.)

Newly synthesized proteins were detected in spermatozoa by incorporating [(35)S]-methionine/cysteine, whereas translation was regulated by 55S mitochondrial ribosomes rather than by 80S cytoplasmic ribosomes, thereby providing evidence of translation during sperm capacitation [16]. Our transcriptomic data indicate that mitochondrial ribosomal subunits, including 39S (large ribosomal subunits, MRPLs), 28S (small ribosomal subunits, MRPSs), and mitoribosome recycling factors, are present in spermatozoa (Supplementary Table S4). While mitochondrial ribosomal protein L48 (MRPL48) was localized at the tip of the acrosome, equatorial region, and tail of spermatozoa (Fig. 4A), MRPL28 was detected in the acrosome and tail of the spermatozoa (Fig. 4B).

Following CP treatment, the abundance of newly synthesized proteins decreased (but was not completely eliminated) during late capacitation (60 – 120 min) in spermatozoa with normal fertility (Fig. 4C, P < 0.05). Furthermore, the intensity of metabolically labeled proteins increased in spermatozoa with reduced fertility at early capacitation, although translation was not inhibited by CP treatment (Fig. 4C). Moreover, dynamic changes in tyrosine-phosphorylated proteins were observed in both normal and reduced fertility spermatozoa during capacitation, while they were arrested following CP treatment during sperm capacitation (Fig. 4D), with no toxic effects (Supplementary Fig. S5).

### Lack of the SPACA1 in the acrosome region by irregular translation leads to reduced fertility

**Fig. 5:**
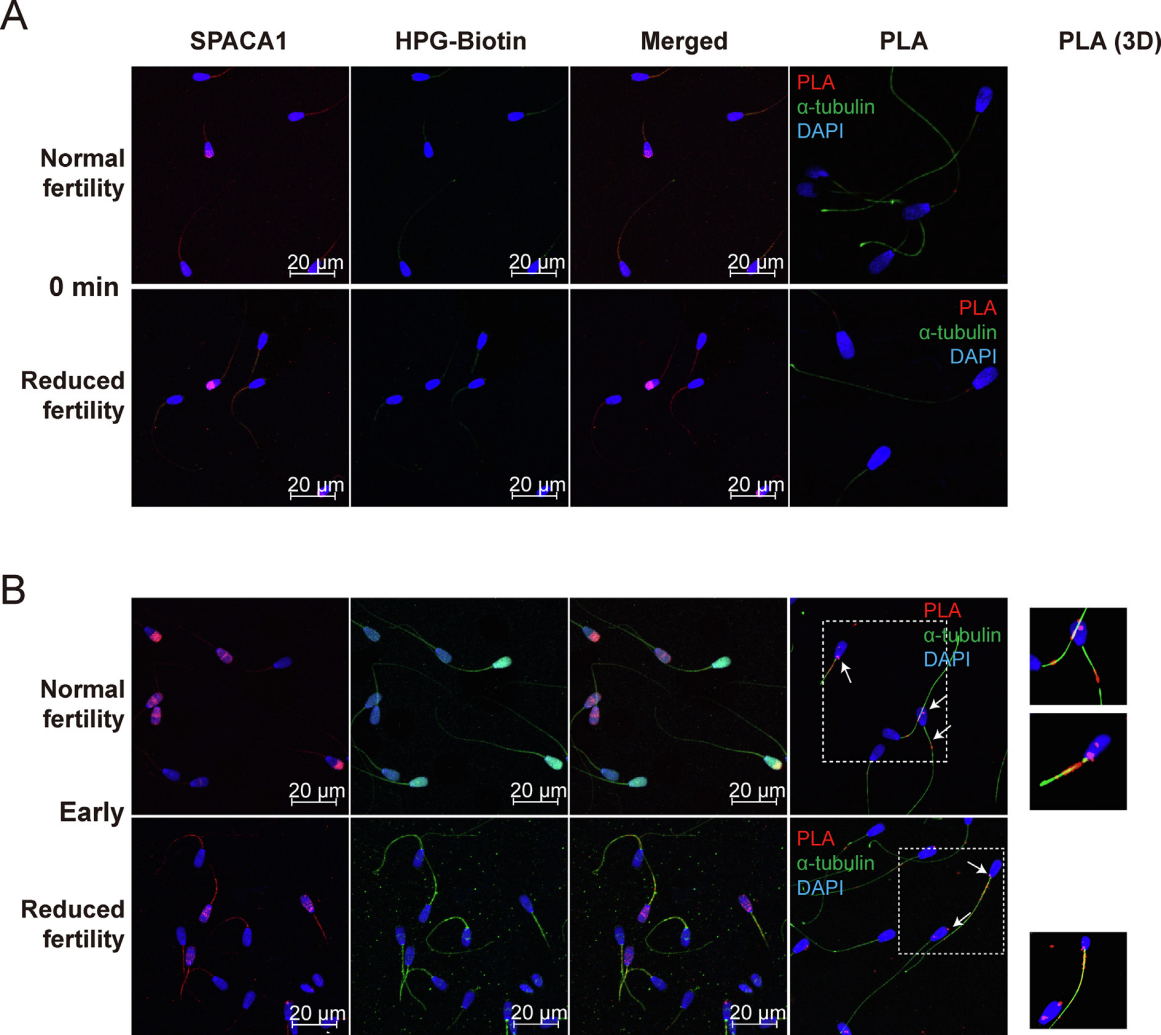

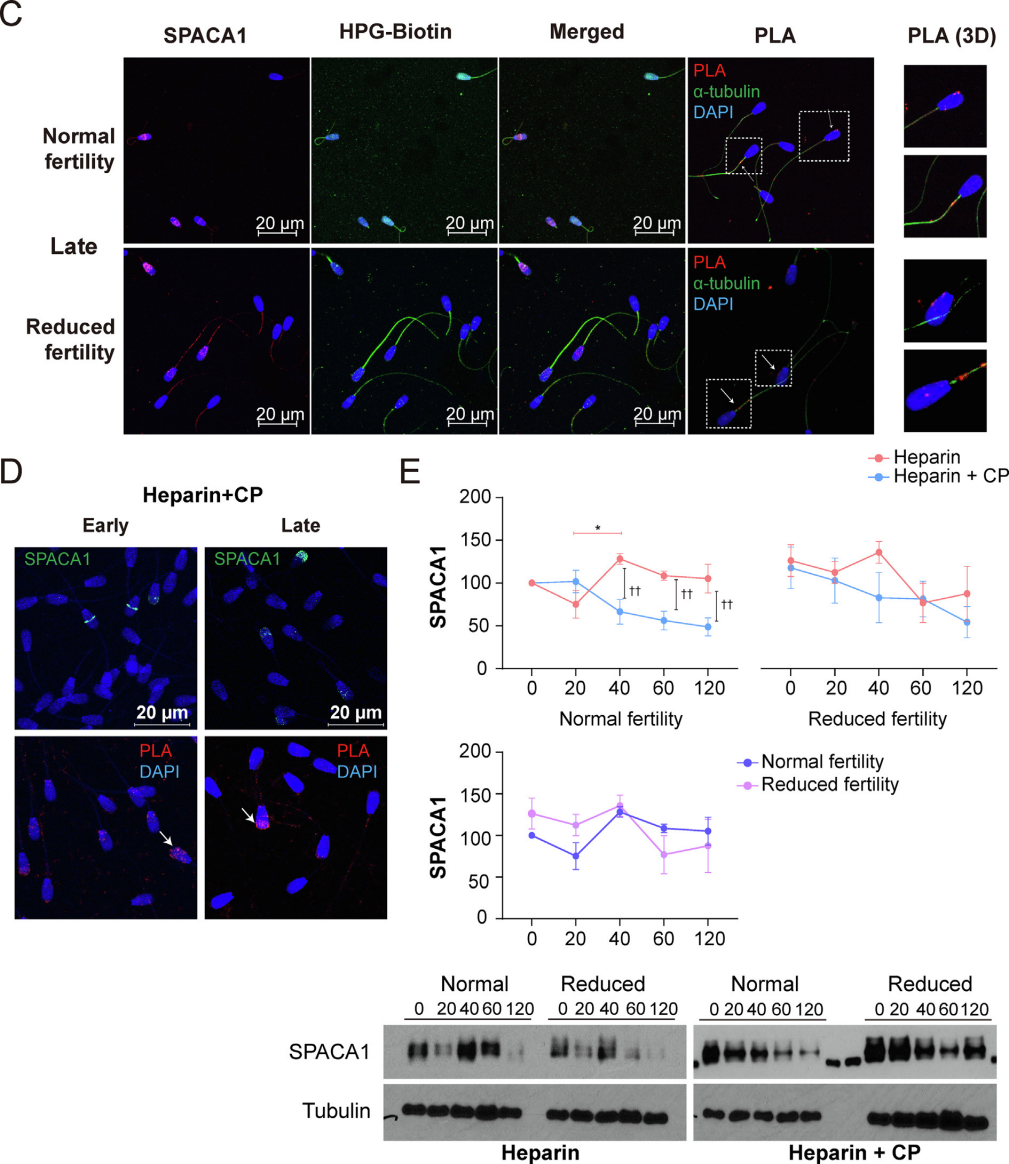
**Distribution changes of newly synthesized SPACA1 following sperm capacitation by FUNCAT-PLA.** Representative immunofluorescence images of spermatozoa from bulls with normal and reduced fertility after staining for SPACA1 (red) and HPG (green). Spermatozoa incubated with 50 µM HPG after (A) 0, (B) 20 (early) and (C) 120 min capacitation (late). FUNCAT–PLA signal (red) from the newly synthesized SPACA1 in spermatozoa and immunostained for α-tubulin (green) as a sperm tail marker. (D) Representative images of SPACA1 (red), HPG (green), and FUNCAT–PLA signals (red) of newly synthesized proteins and SPACA1 in spermatozoa during early and late capacitation with CP. (E) Representative immunoblots probed with antibodies against SPACA1 in normal and reduced fertile spermatozoa during capacitation in the absence (left panel) or presence of CP (right panel). Quantification of SPACA1 protein expression in normal (N, left panel) and reduced (BN, right panel) fertility spermatozoa following capacitation in the absence or presence of CP. *P < 0.05 (n = 3) two-way ANOVA vs. 0 min. †† < 0.01 (n = 3) two-way ANOVA; heparin vs. CP. (For interpretation of the references to colour in this figure legend, the reader is referred to the web version of this article.)

Notably, we also detected various transcripts associated with large and small cytosolic ribosomal protein subunits in spermatozoa (Supplementary Table S4). To detect newly synthesized target proteins directly, a proximity ligation assay (PLA) was performed using an anti-biotin antibody in combination with a targeted protein-specific antibody in the FUNCAT system [26], [28]. The FUNCAT/PLA system allows visualization of the spatial coincidence of newly synthesized proteins and target proteins, which are fertility-dependent differentially expressed proteins during sperm capacitation. Among the six proteins mapped to “Acrosomal vesicles”, three proteins including FAM209A, C7orf61, and SPACA9 were closely interacted (Fig. 2F). Especailly, SPACA proteins are well known as an essential protein for acrosome formation and oocyte fusion [29]. Therefore, SPACA proteins (SPACA1 and SPACA5) were used as the target proteins to FUNCAT/PLA study. Strong fluorescence signals were observed for SPACA1 in the spermatozoa acrosome cap before capacitation, regardless of fertility (Fig. 5A). While newly synthesized proteins were consistently observed in the equatorial and tail regions in reduced fertility spermatozoa, signals were detected in both the tail and entire head in the normal fertility spermatozoa (Fig. 5B). Meanwhile, SPACA1 gradually translocated from the acrosome cap area (0 min, Fig. 5A) to the entire sperm head, with strong signals in the equatorial region in normal fertility at early capacitation (Fig. 5B). While strong SPACA1 signals were detected in the acrosome region in normal fertile spermatozoa at early capacitation, no signals were detected in the acrosome cap of spermatozoa with reduced fertility (Fig. 5B). However, punctate distribution patterns of SPACA1 were observed in spermatozoa with reduced fertility under early capacitation conditions (Fig. 5B), which were comparable to those observed in late capacitated spermatozoa, regardless of fertility (Fig. 5C). The SPACA1–FUNCAT–PLA signal was abundant in the acrosome, neck, and mitochondrial regions of the spermatozoa following early capacitation, regardless of fertility (Fig. 5B). Following late capacitation, a decrease in the SPACA1–FUNCAT–PLA signal was observed in normal fertility spermatozoa, and it was mostly detected in the mitochondrial area rather than in the head of normal fertility spermatozoa (Fig. 5C). Otherwise, the SPACA1–FUNCAT–PLA signal was observed in both the post-equatorial region and mitochondrial part of spermatozoa with reduced fertility following late capacitation (Fig. 5C). Notably, although the SPACA1–FUNCAT–PLA signal was detected in the acrosome region of spermatozoa following CP treatment, the dispersal of SPACA1 throughout the sperm head was suppressed following CP treatment during sperm capacitation, leading to the aberrant localization of SPACA1 in normal fertility spermatozoa (Fig. 5D). The level of SPACA1 expression increased in normal fertility spermatozoa from a capacitation time of 40 min and persisted at a higher level during late capacitation than in non-capacitated spermatozoa. However, upregulation of SPACA1 expression in normal fertility spermatozoa during capacitation was inhibited by CP treatment, indicating that the level of SPACA1 protein expression during capacitation was comparable to that in non-capacitated spermatozoa (Fig. 5E).

### Inhibition of mitochondrial translation delayed translocation of SPACA5 to the acrosome region during capacitation

**Fig. 6:**
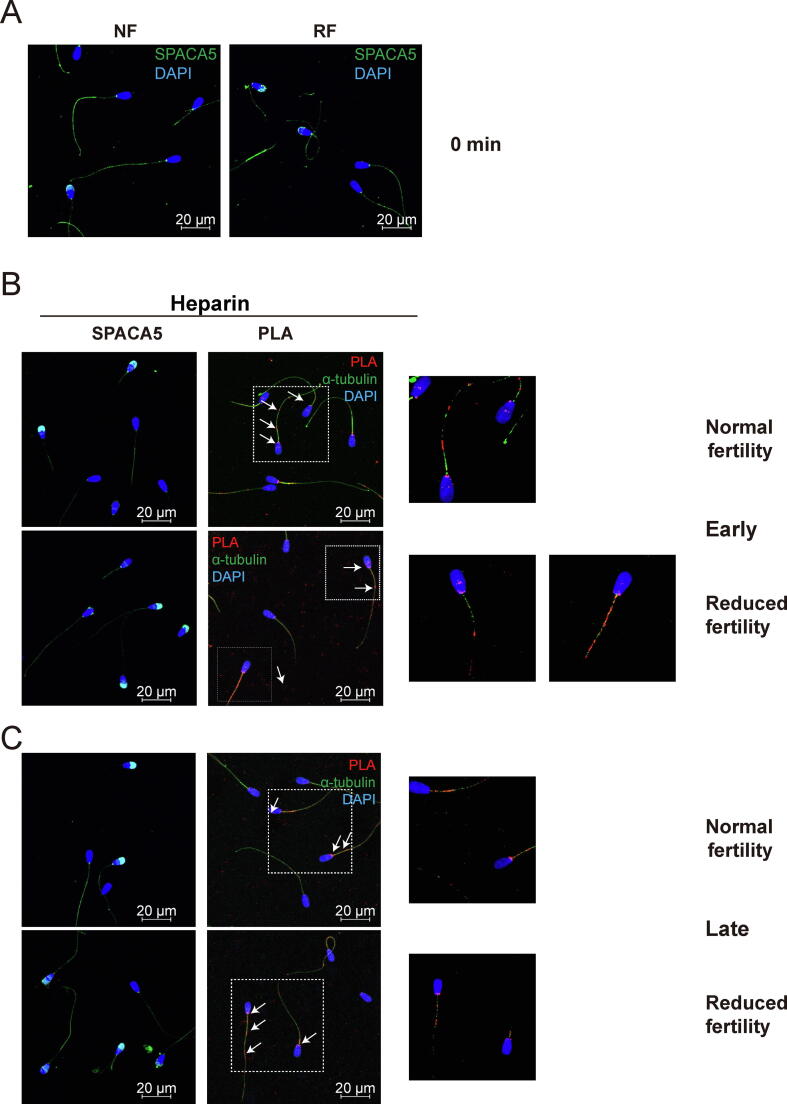

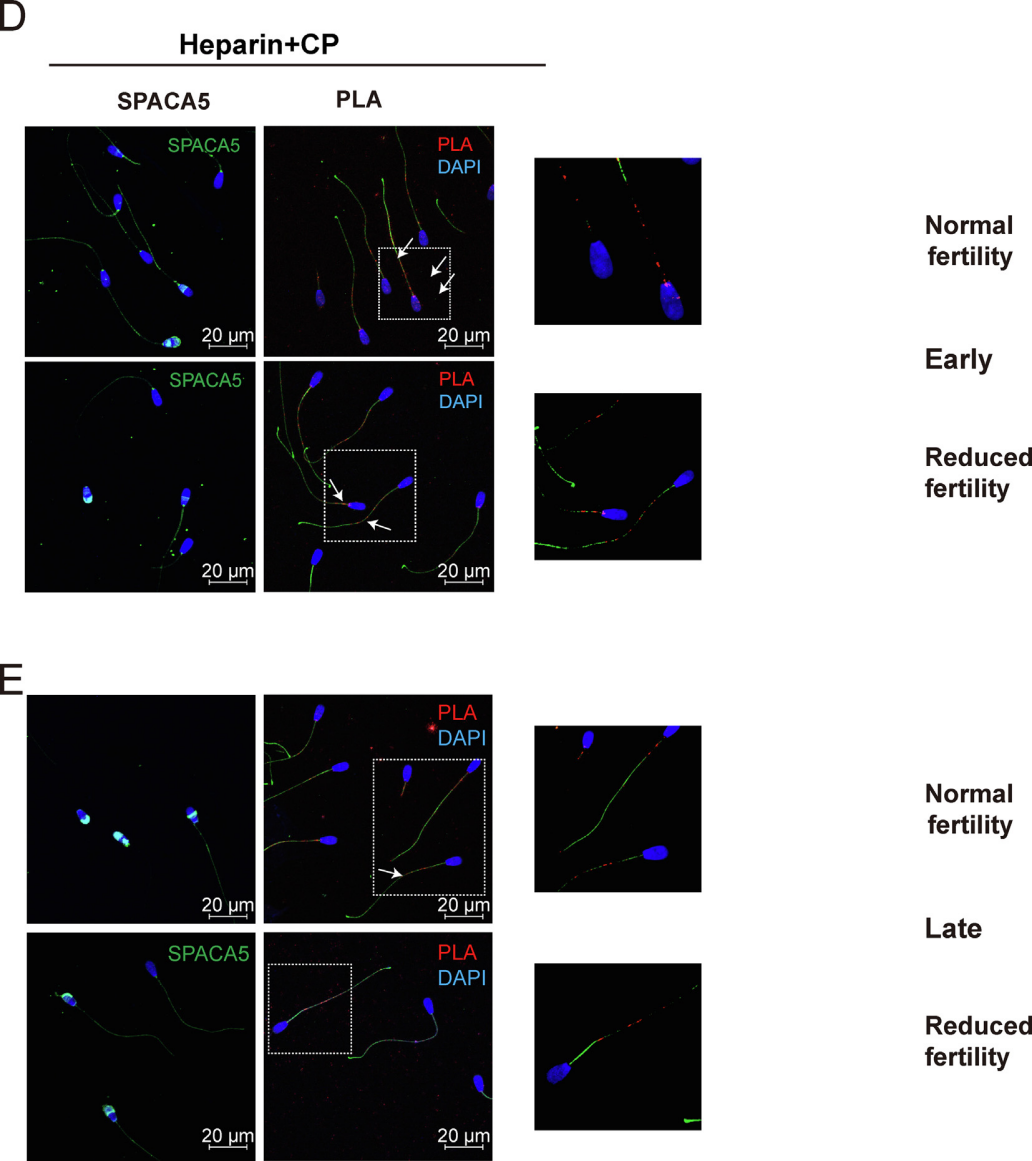
**Distribution changes of newly synthesized SPACA 5 protein following sperm capacitation by FUNCAT-PLA.** Representative immunofluorescence images of spermatozoa from bulls with normal and reduced fertility after staining for SPACA5 (green) and HPG (red). Spermatozoa incubated with 50 µM HPG for (A) 0, (B) 20 (early) and (C) 120 min capacitation (late). FUNCAT–PLA signal (red) from newly synthesized SPACA1 in spermatozoa and immunostained for α-tubulin (green) as a sperm tail marker. (D) Representative images of SPACA1 (red), HPG (green), and FUNCAT–PLA signal (red) from the newly synthesized proteins and SPACA1 in spermatozoa during (D) early and (E) late sperm capacitation in the presence of CP (below panel). α-tubulin (green) was used as a sperm tail marker. (For interpretation of the references to colour in this figure legend, the reader is referred to the web version of this article.)

Robust SPACA5 signals were detected in both the acrosome and principal tail of non-capacitated spermatozoa, regardless of fertility (Fig. 6A). In normal fertility spermatozoa, strong fluorescence signals of SPACA5 were observed consistently in the acrosomal regions and principal tails during capacitation (Fig. 6B and C). The expression patterns of SPACA5 in the spermatozoa with reduced fertility were comparable to those in normal fertility spermatozoa at early capacitation (Fig. 6B). However, the fluorescence signals of SPACA5 were diminished in the acrosome region at late capacitation (Fig. 6C). The SPACA5–FUNCAT–PLA signal was consistently and abundantly detected in the neck and globally on the sperm tail (both in the mitochondria and the principal part) of spermatozoa (Fig. 6B and C), regardless of fertility. Notably, the inhibition of mitochondrial translation by CP treatment during early capacitation led to a loss of SPACA5 in the acrosome and its accumulation in the post-equatorial part, regardless of fertility (Fig. 6D). Although strong SPACA5 signals were detected in the acrosome region in both normal and reduced fertility spermatozoa following late capacitation with CP as well as late capacitation without CP, SPACA5 signals were also observed in the equatorial and post-equatorial regions, which were not detected in late capacitation without CP (Fig. 6E).

## Discussion

Despite recent publications on sperm translation [7], [14], [15], [16], the precise mechanisms involved in the translation of proteins in spermatozoa from capacitation to fertilization and their contribution to fertilization remain unclear. Simultaneously, there is the consensus that a novel method is required to prove whether the protein varies are derived from translation in spermatozoa. Therefore, we expected studies on the molecular basis associated with time-sequentially changes in spermatozoa from spermatogenesis to fertilization to further the in-depth understanding of male fertility complexity [7], [8], [14], [15], [16].

In this study, we used quantitative LC-MS/MS to identify different time-sequential changes in the proteomes of bovine sperm with normal and reduced fertility during sperm capacitation in vitro to recapitulate physiologically relevant changes in spermatozoa. Our data revealed that sperm capacitation was accompanied by dynamic changes in sperm proteomes, in accordance with a previous study [14], in which approximately 50 proteins were upregulated during sperm capacitation in both normal and reduced fertility spermatozoa, whereas more than 70 proteins were downregulated.

GO pathway analysis showing upregulated proteins during sperm capacitation in the reduced fertility are closely related to sperm motility-associated pathways, such as energy-coupled proton transport and the mitochondrial respiratory system. Spermatozoa require extensive energy sources to acquire and maintain the ability to fertilize and perform glycolysis and OXPHOS, both of which actively generate ATP during sperm capacitation [9]. Therefore, a continuously higher level of energy metabolism-related proteins is maintained during sperm capacitation, irrespective of fertility, which may support the energy supply to spermatozoa. Moreover, the activation of mitochondrial energy metabolism is closely related to reactive oxygen species (ROS) production during capacitation. Although moderate levels of ROS are required during capacitation to promote tyrosine phosphorylation, membrane fluidity, and intermolecular interactions in spermatozoa, constant ROS production induces oxidative stress, leading to abnormal function and cell death during sperm capacitation [30]. Protein phosphorylation is a major molecular mechanism with tremendous regulatory and signaling potential associated with various cellular processes, including cell metabolism, proliferation, motility, and cell death [31], [32]. Protein phosphorylation occurs through protein kinases, which add the phosphate group to the protein substrate following the catalysis of adenosine triphosphate (ATP) to adenosine diphosphate (ADP) to transfer the phosphate group [33], [34]. Since the discovery of phosphorylation in spermatozoa, numerous pieces of evidence have suggested that phosphorylation is associated with sperm motility, sperm maturation during transit of the epididymis, capacitation, acrosome reaction in the female reproductive tract, interaction with the zona pellucida, and fertilization of mammalian spermatozoa [35], [36], [37]. However, CP interfered with the upregulation of tyrosine phosphorylation during sperm capacitation and downregulated motion kinematics. These results indicate that upregulated or novo-synthesized proteins accompanying capacitation play essential roles in maintaining sperm functionality and that differences in proteome profiling may lead to dynamic fertility.

During spermiogenesis, the Golgi apparatus produces glycoproteins and proacrosomal vesicles that develop in the acrosome, move to the neck region, and are removed following dynamic morphological changes [38]. The acrosome reaction, a unique exocytotic event in mature spermatozoa, is essential for priming oocyte penetration before fertilization [39]. Several glycoproteins, including glycodelin (PAEP) and lysin, are present in the acrosome region and are released to facilitate the recognition of the extracellular matrix (zona pellucida) in oocytes and trigger fusion between oocytes and spermatozoa [40], [41]. We found that the proteins significantly upregulated in the normal fertility spermatozoa at the early capacitation stage were closely related to the “Golgi to plasma membrane transport” and “cell recognition” signaling pathways, while no changes were detected in the reduced fertility spermatozoa. Given the vital roles of Golgi-derived acrosomal proteins in sperm fertility and oocyte recognition, we hypothesized that the dynamic synthesis of proteins associated with the acrosome reaction occurs during early capacitation, which provides a sequential steady state for the acrosome reaction. Notably, HPG-labeled proteins were detected in the entire head and tail of normal fertility spermatozoa, while strong signals of HPG-labeled proteins were detected in the tail of reduced fertility spermatozoa, similar to the HPG-labeled patterns in fully acrosome-reacted spermatozoa. Moreover, the FUNCAT-PLA assay revealed that the acrosome-associated proteins SPACA1 and SPACA5 co-localize with newly synthesized HPG-labeled proteins. Surprisingly, a relatively earlier loss of acrosomal SPACA1 and SPACA5 was observed in spermatozoa with reduced fertility than in spermatozoa with normal fertility during capacitation, indicating that translation associated with acrosomal proteins may play a crucial role in regulating sperm fertility. These results agree with the results of Zhao et al. [14] which suggested that the proteins associated with the acrosome reaction and sperm-egg fusion are mainly translated during sperm capacitation. Moreover, PCA showed that the proteome profiles of normal and reduced fertility spermatozoa were distinct between the two groups under non-capacitated and early capacitation conditions, whereas those of normal fertility spermatozoa overlapped. This result agrees with the importance of dynamic translation during the initial time of capacitation in promoting the functional capacitation of spermatozoa, which introduces distinct male fertility.

Mitochondria have a separate genetic system, including mtDNA and mitoribosomes, which enables the synthesis of the core subunits of OXPHOS complexes to form functional OXPHOS enzyme complexes with nuclear-encoded subunits [18], [19]. In addition to functioning as energetic machinery, mitochondria contribute to proteostasis through the regulation of cytosolic translation and dissolution of damaged proteins under stress conditions [20], [21], [22]. Following severe heat shock stress, mitochondria-specific MRPL18 is alternatively translated into the cytosolic isoform of MRPL18, which is integrated into an 80S ribosome complex to produce heat shock stress-associated proteins such as HSP 70 [22]. A previous study by Gur and Breitbart [16] demonstrated that 55S mitoribosomes are actively involved in nuclear-encoded protein translation during sperm capacitation rather than 80S cytosolic ribosomes. Here, we found that the inhibition of mitochondrial translation by CP did not completely diminish protein synthesis during sperm capacitation. Moreover, CP treatment delayed the synthesis and translocation of acrosomal proteins during capacitation and altered the localization of protein synthesis. Based on these results, we speculate that MRPs directly participate in protein synthesis during sperm capacitation as isoforms of cytosolic ribosomal proteins. Moreover, the presence of large and small cytosolic ribosomal subunits in spermatozoa suggests that de novo protein synthesis could be regulated by both mitochondrial and cytosolic ribosomal proteins. The upregulation of these proteins during capacitation may indicate normal fertility, supporting capacitation, acrosome reaction, fertilization, and subsequent processes.

Our spatiotemporal analysis of protein expression during sperm capacitation in bulls with normal and reduced fertility provides a global picture that reveals sequential changes in sperm proteins during capacitation. Moreover, it reveals different phenomena in acrosomal protein changes according to sperm fertility, which can provide insights into the capacitation–fertility interaction. We also optimized the FUNCAT/PLA system in spermatozoa to allow visualization of the spatial coincidence of newly synthesized and target proteins. We demonstrated that short-term application of HPG (20 min), followed by a click reaction with an azide-bearing fluorescent tag, gives rise to clearly detectable signals in spermatozoa. Optimization of the protocol to detect newly synthesized proteins in spermatozoa showed the specific compartment where the translation site is located for a better understanding of both capacitation-related biological mechanisms and proteome plasticity during capacitation. Interestingly, newly synthesized proteins were observed from early capacitation conditions (within 20 min) to the acrosome reaction (2 h) but were not detected in fully acrosome-reacted spermatozoa. This result emphasizes that a translation study using early capacitation conditions is essential because acrosome-reacted spermatozoa undergo long-term incubation loss of their membrane proteins, which could complicate the translation evidence associated with spermatozoa during capacitation.

Spermatozoa in the female reproductive tract are considered invaders and are exposed to various challenges during their journey to fertilization. After overcoming obstacles on the way to the egg, less than 0.01 % of spermatozoa encounter oocytes [42]. These capitalized spermatozoa are evolutionarily selected by outcompeting other spermatozoa to achieve their goal of fertilization, which can produce offspring with enhanced fertilizing ability [43], [44]. In non-human animals, multivariate approaches, such as male-male competition based on multiple female mating systems in animal environments, have been used to elucidate the evolutionary mechanisms of sperm quality [45], [46]. Regarding the dynamic changes in the spermatozoa present in the female reproductive tract before fertilization, an understanding of the selective forces that influence the evolution of sperm functional changes in the female reproductive tract that contribute to sperm fertility is required. However, studies on female-sperm interactions have focused on the role of the female reproductive tract rather than spermatozoa, such as the establishment of barriers for reducing the number of spermatozoa reaching the oocyte and restriction of multiple sperm penetration into the oocytes [47], [48]. Therefore, we anticipate that assessing the relationship between dynamic sperm translation during capacitation and sperm fertility will enhance our understanding of how protein modifications influence the evolution of sperm quality.

## Conclusion

We believe that this is the first indication of different spatiotemporal regulation in the spermatozoa translation system during sperm capacitation, which is related to male fertility. Although it is unclear how translation initiation is regulated during sperm capacitation, we suggest that time-sequential microenvironmental changes in sperm proteomes during capacitation may lead to the orchestration of proteins that complete fertilization. Moreover, this advanced understanding of sperm cell biology demonstrates a sensitive and adequate response of sperm populations to capacitation stimuli in a timely manner, which is essential for fertilization. To the best of our knowledge, there are only a few studies that focus on the pathological translation during capacitation that causes male infertility. Therefore, we believe that this study provides direct evidence that the loss of on-time translation of acrosome-associated proteins during early capacitation may lead to an irregular acrosome reaction and delayed priming and penetration of oocytes, resulting in a reduction in male fertility. Aberrant upregulation of acrosomal proteins may lead to an irregular, delayed, or premature acrosome reaction. We identified specific proteins that represent slow upregulation during capacitation in spermatozoa with reduced fertility, which can be used as candidate biomarkers for the prediction of sperm fertility defects. Based on our results, we believe that this study may accelerate the development of male contraceptive methods or therapeutic methods for male infertility by screening antagonists or agonists that regulate aberrant sperm translation depending on the stage of fertilization.

## Compliance with Ethics Requirements

*All Institutional and National Guidelines for the care and use of animals (fisheries) were followed.*

All animal experiments were approved by the Institutional Animal Care and Use Committee of Chung-Ang University, Seoul, Korea (IACUC Number: 2016-00009).

## CRediT Author Statement

**Yoo-Jin Park:** conceptualization, data curation, investigation, validation, visualization, writing, and funding acquisition. **Won-Ki Pang:** formal analysis, data curation, and investigation. **Do-Yeal Ryu**: investigation, methodology, and data analysis. **Md Saidur Rahman**: conceptualization, experiments, and data curation. **Myung-Geol Pang**: conceptualization, resources, supervision, and funding acquisition. All of the authors edited the manuscript.

## Fundings

Basic Science Research Program through the National Research Foundation of Korea (NRF) funded by the Ministry of Education (NRF-2018R1A6A1A03025159).

Basic Science Research Program through the National Research Foundation of Korea (NRF)(RS-2024-00343755).

Funding for open access charge: Ministry of Education

## Declaration of competing interest

The authors declare that they have no known competing financial interests or personal relationships that could have appeared to influence the work reported in this paper.
