# Supplementary material for: Spatiotemporal translation of sperm acrosome associated proteins during early capacitation modulates sperm fertilizing ability
Source: J Adv Res. 2025 Mar 18;79:313–29. doi: 10.1016/j.jare.2025.03.035 (PMC12766180; doi:10.1016/j.jare.2025.03.035)
Supplement: Supplementary Data 1 [file mmc1.pdf]

Supplemental Figure Legends

Figure S1. (A) The average fertility rate in spermatozoa and capacitation status following capacitation. A The average fertility rate of normal (blue) and reduced fertility (pink) spermatozoa. Data represent the mean ± S.E.M. (n = 5). ***Significant differences between normal and reduced fertility spermatozoa ($p < 0.001$). (B) Different patterns of chlortetracycline fluorescence staining, such as, capacitated, acrosome-reacted, and non-capacitated spermatozoa following capacitation.

Figure S2. Pearson correlation plots of biological replicates for capacitated spermatozoa between normal and reduced fertility.

Figure S3. Quantitative analysis of capacitation associated proteins. Comparison of proteins from normal and reduced fertility spermatozoa during (A) early and (B) late capacitation. log2 fold change (X axis) and p value ≤ 0.05.

Figure S4. Representative image of HPG-labelled proteins in spermatozoa according to the status of the intact acrosome following 2 h capacitation by transmission electron microscopy.

Figure S5. Effect of CP treatment during sperm capacitation on lactate dehydrogenase levels in spermatozoa.

Supplemental Tables

Table S1. Comparison of sperm motility and motion kinematics between normal and reduced fertility spermatozoa during capacitation.

		**Heparin**		**Heparin + CP**	
	**Incubation time**	**Normal Fertility**	**Reduced Fertility**	**Normal Fertility**	**Reduced Fertility**
Motility (%)	0 min	73.49 ± 1.09	73.63 ± 2.90	73.49 ± 1.09	73.63 ± 2.90
	20 min	72.33 ± 2.34	68.95 ± 1.69	63.39 ± 0.90	54.23 ± 4.08**
	40 min	56.63 ± 4.96*	46.95 ± 2.95***	51.72 ± 2.78***	47.37 ± 5.82***
	60 min	52.14 ± 5.71**	40.81 ± 5.02***	44.62 ± 2.88***	41.48 ± 5.30***
	120 min	42.91 ± 3.24***	37.12 ± 1.95***	35.77 ± 1.95***	27.83 ± 4.43***
VCL (μm/s)	0 min	94.52 ± 0.80	97.95 ± 2.20	94.52 ± 0.80	97.95 ± 2.20
	20 min	101.21 ± 3.00	99.95 ± 2.61	81.73 ± 3.37$^{*,\#}$	84.51 ± 2.40$^{*,\#}$
	40 min	106.86 ± 4.20	106.68 ± 1.66	83.44 ± 2.69$^{***,\#\#\#}$	87.91 ± 4.71$^{\#}$
	60 min	110.09 ± 0.29*	109.61 ± 5.55	85.01 ± 4.55$^{\#\#\#}$	89.34 ± 5.07$^{\#\#\#}$
	120 min	110.27 ± 2.53**	101.02 ± 4.43	88.54 ± 1.07$^{\#\#\#}$	86.27 ± 2.27$^{\#}$
VSL (μm/s)	0 min	62.61 ± 0.87	62.02 ± 0.86	62.61 ± 0.87	62.02 ± 0.86
	20 min	74.05 ± 4.07	73.26 ± 2.49	56.03 ± 2.97$^{\#}$	57.53 ± 1.15$^{\#}$
	40 min	83.97 ± 4.95***	81.84 ± 3.15***	58.76 ± 2.88$^{\#\#\#}$	60.31 ± 4.08$^{\#\#\#}$
	60 min	85.64 ± 2.16***	84.07 ± 6.62***	62.64 ± 3.02$^{\#\#\#}$	64.39 ± 3.70$^{\#\#}$
	120 min	86.97 ± 4.06***	80.70 ± 4.98***	65.15 ± 0.73$^{\#\#\#}$	67.49 ± 2.05
VAP	0 min	66.46 ± 0.91	65.99 ± 1.41	66.46 ± 0.91	65.99 ± 1.41
	20 min	75.23 ± 3.26	73.56 ±1.97	57.10 ± 2.64$^{\#\#}$	59.00 ± 1.46$^{\#}$
	40 min	83.48 ± 4.31**	81.51 ± 2.36**	59.58 ± 2.87$^{\#\#\#}$	61.59 ± 4.13$^{\#\#\#}$
	60 min	84.89 ± 2.44**	84.48 ± 5.39***	62.76 ± 3.28$^{\#\#\#}$	64.77 ± 3.60$^{\#\#\#}$
	120 min	86.45 ± 2.92***	80.35 ± 4.99**	66.03 ± 1.12$^{\#\#\#}$	67.06 ± 1.83
LIN	0 min	66.24 ± 1.24	63.39 ± 1.22	66.24 ± 1.24	63.39 ± 1.22
	20 min	73.07 ± 2.79	73.24 ±0.97***	68.54 ± 2.04	68.18 ± 1.27
	40 min	78.54 ± 1.68***	76.60 ± 1.93***	70.08 ± 1.15$^{\#}$	68.43 ± 1.10$^{\#}$
	60 min	77.82 ± 2.19***	76.33 ± 2.25***	73.66 ± 0.68*	71.98 ± 0.87**
	120 min	78.73 ± 2.18***	79.82 ± 2.82***	73.56 ± 1.00*	78.13 ± 2.12***
ALH (μm)	0 min	4.95 ± 0.03	4.99 ± 0.11	4.95 ± 0.03	4.99 ± 0.11
	20 min	5.37 ± 0.16	5.29 ± 0.13	4.27 ± 0.15$^{*,\#\#}$	4.50 ± 0.12$^{\#}$
	40 min	5.78 ± 0.26*	5.71 ± 0.11*	4.32 ± 0.15$^{\#\#\#}$	4.61 ± 0.23$^{\#\#\#}$
	60 min	5.91 ± 0.07**	5.97 ± 0.30**	4.49 ± 0.21$^{\#\#\#}$	4.73 ± 0.24$^{\#\#\#}$
	120 min	5.97 ± 0.16***	5.53 ± 0.23***	4.72 ± 0.16$^{\#\#\#}$	4.68 ± 0.12$^{\#}$

Table S2. Upregulated proteins of spermatozoa during early capacitation.

Normal fertility		Reduced fertility	
**Accession No.**	**Gene Name**	**Accession No.**	**Gene ID**
A0A3Q1LHA3	Acyl-CoA dehydrogenase short chain	A0A3Q1LF83	RING1 and YY1 binding protein
A0A3Q1MQG7	NADH dehydrogenase [ubiquinone] 1 beta subcomplex subunit 8, mitochondrial (Complex I-ASHI) (NADH-ubiquinone oxidoreductase ASHI subunit)	A0A3Q1MDV9	Rhophilin associated tail protein 1 like
A0A3Q1MMP2	SAMM50 sorting and assembly machinery component	F1MRI2	Mitochondrial import inner membrane translocase subunit Tim21
A0A3Q1MGZ4	Triokinase/FMN cyclase (EC 4.6.1.15) (Bifunctional ATP-dependent dihydroxyacetone kinase/FAD-AMP lyase (cyclizing))	G3X8G1	ATP6V1F neighbor
A0A3Q1NJR8	Antithrombin-III (Serpin C1)	A0A3Q1M4Y2	Cilia and flagella associated protein 57
A0A0A0MP88	Serpin peptidase inhibitor, clade A (alpha-1 antiproteinase, antitrypsin), member 3	P33097	Aspartate aminotransferase, cytoplasmic (cAspAT) (EC 2.6.1.1) (EC 2.6.1.3) (Cysteine aminotransferase, cytoplasmic) (Cysteine transaminase, cytoplasmic) (cCAT) (Glutamate oxaloacetate transaminase 1) (Transaminase A)
A0A3Q1MVL5	oxoglutarate dehydrogenase (succinyl-transferring) (EC 1.2.4.2)	A0A3Q1LY08	Fumarate hydratase, mitochondrial (EC 4.2.1.2)
A0A3Q1M5Z8	palmitoyl-protein hydrolase (EC 3.1.2.22)	Q32LC5	CKLF-like MARVEL transmembrane domain containing 2
A0A3Q1LW84	Protein phosphatase 2 scaffold subunit Aalpha	F1N594	Radial spoke head 14 homolog
A0A3Q1LTJ2	Doublecortin domain containing 2C	Q5XQN5	Keratin, type II cytoskeletal 5 (Cytokeratin-5) (CK-5) (Keratin-5) (K5) (Type-II keratin Kb5)
F1MQJ0	Angiotensin-converting enzyme (ACE) (EC 3.4.15.1) (Dipeptidyl carboxypeptidase I) (Kininase II) (CD antigen CD143) [Cleaved into: Angiotensin-converting enzyme, soluble form]	A0A3Q1MDH5	Acetyl-CoA acyltransferase 2
P15497	Apolipoprotein A-I (Apo-AI) (ApoA-I) (Apolipoprotein A1) [Cleaved into: Proapolipoprotein A-I (ProapoA-I); Truncated apolipoprotein A-I]	A0A3Q1LFR8	Dynein axonemal heavy chain 17
A0A3Q1LPJ9	ADAM metallopeptidase domain 20	A0A3Q1MR22	Desmoplakin
F1MMT9	Ferritin	Q2HJ86	Tubulin alpha-1D chain (EC 3.6.5.-) [Cleaved into: Detyrosinated tubulin alpha-1D chain]
A0A3Q1LHW2	Ankyrin repeat domain 42	E1BFM2	Serine protease 50
A0A3Q1MQ86	Zona pellucida binding protein 2	E1BJL9	Cilia- and flagella- associated

			protein 210
A0A3Q1LYE8	Progestagen-associated endometrial protein	A0A3Q1M8K4	Integrin subunit alpha 6
F1N2N9	Outer dynein arm-docking complex subunit 1	A0A452DIW4	Malate dehydrogenase (EC 1.1.1.37)
Q02369	NADH dehydrogenase [ubiquinone] 1 beta subcomplex subunit 9 (Complex I-B22) (CI-B22) (NADH-ubiquinone oxidoreductase B22 subunit)	G3N284	Actin related protein T1
A0A3Q1M7X9	Acyl-CoA thioesterase 9	E1B7Q2	Adenylate kinase 7
A0A3Q1MYR8	Keratin 3	A0A3Q1M6U5	Protein-serine/threonine kinase (EC 2.7.11.-)
A0A452DIK3	NADH dehydrogenase [ubiquinone] iron-sulfur protein 8, mitochondrial (Complex I-23kD) (NADH-ubiquinone oxidoreductase 23 kDa subunit)	A0A3Q1MKC8	Keratin 7
F1MYG5	Lamin A/C	F6RLA2	Testis specific serine kinase 4
P08904	Ribonuclease K6 (RNase K6) (EC 3.1.27.-) (K6b) (Ribonuclease K2) (RNase K2)	A0A3Q1M1M7	Junction plakoglobin
A0A3Q1LKP0	Peroxisome proliferator activated receptor delta	A0A3Q1N5Z4	Ig-like domain-containing protein
P02722	ADP/ATP translocase 1 (ADP,ATP carrier protein 1) (ADP,ATP carrier protein, heart isoform T1) (Adenine nucleotide translocator 1) (ANT 1) (Solute carrier family 25 member 4)	F1ME99	Centrosomal protein 135
F6RLA2	Testis specific serine kinase 4	E1BCP9	Galactosidase beta 1 like
A0A3Q1LZ21	Hydroxysteroid dehydrogenase like 2	Q02365	NADH dehydrogenase [ubiquinone] 1 beta subcomplex subunit 3 (Complex I-B12) (CI-B12) (NADH-ubiquinone oxidoreductase B12 subunit)
Q3T0R7	3-ketoacyl-CoA thiolase, mitochondrial (EC 2.3.1.16) (Acetyl-CoA acetyltransferase) (EC 2.3.1.9) (Acetyl-CoA acyltransferase) (Acyl-CoA hydrolase, mitochondrial) (EC 3.1.2.-, EC 3.1.2.1, EC 3.1.2.2) (Beta-ketothiolase) (Mitochondrial 3-oxoacyl-CoA thiolase)	P61285	Dynein light chain 1, cytoplasmic (Dynein light chain LC8-type 1)
Q0II87	Transcription factor A, mitochondrial (mtTFA)	A0A3Q1LS67	Enoyl-CoA hydratase 1
A0A140T860	Calcineurin like phosphoesterase domain containing 1	A0A3Q1LZL0	Dynein axonemal heavy chain 8
Q32LJ7	RIB43A-like with coiled-coils protein 2	A0A3S5ZPN7	Coiled-coil domain containing 116
F1N3A8	Chromosome 27 C4orf47 homolog	A0A3Q1LL25	Serine/threonine-protein phosphatase 2A activator (EC 5.2.1.8) (Phosphotyrosyl phosphatase activator)
A0A3Q1M8K4	Integrin subunit alpha 6	F1MMY0	SSD domain-containing protein
A0A3Q1MVR8	Multifunctional fusion protein [Includes: Delta-1-pyrroline-5-	A0A3Q1LRV1	Coiled-coil domain containing 183

	carboxylate dehydrogenase (P5C dehydrogenase) (L-glutamate gamma-semialdehyde dehydrogenase); L-glutamate gamma-semialdehyde dehydrogenase (EC 1.2.1.88)]		
A0A3Q1MXT9	Reverse transcriptase domain-containing protein	F1N3A8	Chromosome 27 C4orf47 homolog
A0A3Q1LR88	F-actin-capping protein subunit beta	Q58DG1	MYG1 exonuclease (EC 3.1.-.-)
A6QQ16	NSL1 component of MIS12 kinetochore complex (NSL1 protein)	A0A3Q1MWG7	Malic enzyme
Q3ZCK2	RAS like proto-onco A (V-ral simian leukemia viral oncogene homolog A (Ras related))	A0A3Q1LRC3	Cilia and flagella associated protein 43
G3N0H2	Chromosome 11 C2orf16 homolog	A4FV54	Ras-related protein Rab-8A (EC 3.6.5.2)
E1BIF7	SH3 domain-binding protein 5 (SH3BP-5)	F1MK41	Short chain dehydrogenase/reductase family 39U member 1
Q5XQN5	Keratin, type II cytoskeletal 5 (Cytokeratin-5) (CK-5) (Keratin-5) (K5) (Type-II keratin Kb5)	F1MQJ0	Angiotensin-converting enzyme (ACE) (EC 3.4.15.1) (Dipeptidyl carboxypeptidase I) (Kininase II) (CD antigen CD143) [Cleaved into: Angiotensin-converting enzyme, soluble form]
A0A3Q1ND53	Hydroxyacylglutathione hydrolase	A0A3Q1M3S4	Septin-7
A0A3Q1MBV5	X-prolyl aminopeptidase 3	A0A3Q1MIR4	Centrosomal protein of 44 kDa
A0A3Q1LUL6	Carboxymuconolactone decarboxylase-like domain-containing protein	A0A3Q1LHW9	Coilin
E1BFM2	Serine protease 50	A0A3Q1LUL6	Carboxymuconolactone decarboxylase-like domain-containing protein
P11024	NAD(P) transhydrogenase, mitochondrial (EC 7.1.1.1) (Nicotinamide nucleotide transhydrogenase) (Pyridine nucleotide transhydrogenase)	A1L595	Keratin, type I cytoskeletal 17 (Cytokeratin-17) (CK-17) (Keratin-17) (K17)
Q4R0H2	Spermadhesin 2	E1BCN8	Phosphoethanolamine/phosphocholine phosphatase 1
P05632	ATP synthase subunit epsilon, mitochondrial (ATPase subunit epsilon) (ATP synthase F1 subunit epsilon)	A0A3Q1MEH5	Coiled-coil domain containing 63
A0A452DIK0	NADH dehydrogenase [ubiquinone] 1 alpha subcomplex subunit 12	A0A3Q1LXK7	Cytochrome c oxidase subunit 6C (Cytochrome c oxidase polypeptide VIc)
A0A452DIM5	Cytochrome c oxidase subunit (Cytochrome c oxidase polypeptide VIa)	E1BCN4	AMP-dependent synthetase/ligase domain-containing protein
Q0V8B1	Protein Flattop (Cilia- and flagella-associated protein 126)	Q02369	NADH dehydrogenase [ubiquinone] 1 beta subcomplex subunit 9 (Complex I-B22) (CI-B22) (NADH-ubiquinone oxidoreductase B22 subunit)
G3X7W8	Keratin 16	F1MES6	UBX domain protein 6

F1MZD5	Dehydrogenase/reductase (SDR family) member 4	A0A452DIM5	Cytochrome c oxidase subunit (Cytochrome c oxidase polypeptide VIa)
Q6QTG5	Cytochrome c oxidase subunit 3	F1MZB5	Protein SPMIP9 (Sperm microtubule inner protein 9) (Testis-expressed sequence 37 protein) (Testis-specific conserved protein of 21 kDa)
Q5DPW9	Cystatin E/M	Q0II40	Dual specificity phosphatase 21 (Similar to Dual specificity protein phosphatase 18 (Low molecular weight dual specificity phosphatase 20))
Q32P93	Glycosylphosphatidylinositol anchored molecule like (Glycosylphosphatidylinositol anchored molecule like protein)	A0A3Q1LUP6	POC1 centriolar protein B
Q32KZ3	Leucine rich repeat containing 18	A0A3Q1LXS2	Cilia and flagella associated protein 161
G3MWG4	isoleucine--tRNA ligase (EC 6.1.1.5) (Isoleucyl-tRNA synthetase)	P81019	Seminal plasma protein BSP-30 kDa (BSP-30K)
F1MY93	Leucine rich repeat containing 34	A0A3Q1LW70	Adenylate kinase 8 (EC 2.7.4.6)
F1MR53	Coiled-coil domain containing 27	A0A3Q1M9N4	AU RNA binding methylglutaconyl-CoA hydratase
F1MP48	Chaperonin containing TCP1 subunit 6B	Q2HJ54	Phosphatidylinositol transfer protein alpha isoform (PI-TP-alpha) (PtdIns transfer protein alpha) (PtdInsTP alpha) (Phosphatidylinositol-transfer protein 35 kDa isoform) (PI-TP 35 kda isoform)
F1MMA3	Testis expressed 55	F1MP48	Chaperonin containing TCP1 subunit 6B
F1MBU8	Dpy-19 like 2	A0A3Q1MUU9	T-complex protein 1 subunit eta (TCP-1-eta) (CCT-eta)
E1BLT3	Spectrin beta, non-erythrocytic 4	A0A3Q1MPF1	Rab GDP dissociation inhibitor
E1BKQ1	Sulfotransferase (EC 2.8.2.-)	Q32L77	Cilia- and flagella-associated protein 95
E1BCN8	Phosphoethanolamine/phosphocholine phosphatase 1	A0A3Q1LW84	Protein phosphatase 2 scaffold subunit Aalpha
E1BCN4	AMP-dependent synthetase/ligase domain-containing protein	Q2YDE4	Proteasome subunit alpha type-6
E1B8W3	Outer dynein arm-docking complex subunit 2 (Armadillo repeat-containing protein 4)	A0A3Q1MZ77	CutA divalent cation tolerance homolog
E1B8R6	RBR-type E3 ubiquitin transferase (EC 2.3.2.31)	A0A3Q1ND53	Hydroxyacylglutathione hydrolase
E1B7I3	ADAM metallopeptidase domain 20	Q0II90	Protein FAM81B
A8YXY4	NADH:ubiquinone oxidoreductase subunit S6 (NDUFS6 protein)	A0A3Q1MCL0	Follistatin like 5
A0A3S5ZPI7	Cytochrome b5 type B	A0A3Q1LFK7	Piercer of microtubule wall 2 protein
A0A3Q1MUN0	Sorbitol dehydrogenase (SDH) (XDH) (EC 1.1.1.14) (EC 1.1.1.9) (L-iditol 2-dehydrogenase) (Polyol dehydrogenase) (Xylitol	Q2TBS5	Serine peptidase inhibitor, Kazal type 2 (Acrosin-trypsin inhibitor) (Serine peptidase inhibitor, Kazal type 2 B)

	dehydrogenase)		
A0A3Q1MT14	Sperm microtubule inner protein 11 (Testis-expressed protein 49)	A4IFP2	KRT4 protein (Keratin 4)
A0A3Q1MPF1	Rab GDP dissociation inhibitor	A6QLZ6	GLRX5 protein (Glutaredoxin 5)
A0A3Q1ML84	ATP synthase membrane subunit K, mitochondrial	Q2T9S0	Tubulin beta-3 chain
A0A3Q1MIR4	Centrosomal protein of 44 kDa	A0A3Q1M3C8	H1.7 linker histone
A0A3Q1MFE4	MICOS complex subunit MIC60 (Mitofilin)	A0A3Q1LYE8	Progestagen-associated endometrial protein
A0A3Q1MEM6	PWWP domain containing 3A, DNA repair factor	A0A3Q1LPJ9	ADAM metallopeptidase domain 20
A0A3Q1M9N4	AU RNA binding methylglutaconyl-CoA hydratase	A0A3Q1M3M6	D-ribitol-5-phosphate cytidylyltransferase (EC 2.7.7.40) (2-C-methyl-D-erythritol 4-phosphate cytidylyltransferase-like protein) (Isoprenoid synthase domain-containing protein)
A0A3Q1M9F3	Nucleoporin 155	Q148H7	Keratin, type II cytoskeletal 79 (Cytokeratin-79) (CK-79) (Keratin-79) (K79) (Type-II keratin Kb38)
A0A3Q1M6U5	Protein-serine/threonine kinase (EC 2.7.11.-)	P34933	Heat shock-related 70 kDa protein 2 (Heat shock 70 kDa protein 3) (HSP70.3)
A0A3Q1M6F8	UBX domain protein 11	A0A3Q1MQ86	Zona pellucida binding protein 2
A0A3Q1M4Y2	Cilia and flagella associated protein 57	Q148D3	Fumarate hydratase, mitochondrial (EC 4.2.1.2)
A0A3Q1M3S4	Septin-7	P67868	Casein kinase II subunit beta (CK II beta) (Phosvitin)
A0A3Q1M2L7	Testis expressed 46	E1B9R5	Dynein axonemal heavy chain 8
A0A3Q1LUJ9	Glycerophosphodiester phosphodiesterase 1	A0A0A0MP88	Serpin peptidase inhibitor, clade A (alpha-1 antiproteinase, antitrypsin), member 3
A0A3Q1LTC5	Dynein regulatory complex subunit 3	E1BHQ2	Selenoprotein V
A0A3Q1LTA7	Myeloid leukemia factor 1	E1B9I5	Cilia- and flagella-associated protein 276
A0A3Q1LS67	Enoyl-CoA hydratase 1	E1BHZ3	FAM161 centrosomal protein A
A0A3Q1LRV1	Coiled-coil domain containing 183	A0A3Q1M842	Solute carrier family 2, facilitated glucose transporter member 5 (Fructose transporter) (Glucose transporter type 5, small intestine)
A0A3Q1LL25	Serine/threonine-protein phosphatase 2A activator (EC 5.2.1.8) (Phosphotyrosyl phosphatase activator)	A8E4N3	Radial spoke head protein 3 homolog (A-kinase anchor protein RSPH3) (Radial spoke head-like protein 2)
A0A3Q1LK47	AFG3 like matrix AAA peptidase subunit 2	A0A3Q1LV18	T-complex protein 1 subunit beta (CCT-beta)
A0A3Q1LJL3	Dynamin-like 120 kDa protein, mitochondrial (EC 3.6.5.5)	E1BPM9	Dynein axonemal intermediate chain 2
A0A3Q1LJI4	[tau protein] kinase (EC 2.7.11.26)	P05632	ATP synthase subunit epsilon, mitochondrial (ATPase subunit epsilon) (ATP synthase F1 subunit epsilon)
A0A3Q1LJ59	ABC transporter domain-containing protein	A0A3Q1ML84	ATP synthase membrane subunit K, mitochondrial
A0A3Q1LFK7	Piercer of microtubule wall 2 protein	Q2KJD2	Vesicle-associated membrane protein 3 (VAMP-3) (Synaptobrevin-3)

A0A140T871	glutamate dehydrogenase [NAD(P)(+)] (EC 1.4.1.3)	G3MZU8	Transmembrane protein 210
Q5E987	Proteasome subunit alpha type-5	P00442	Superoxide dismutase [Cu-Zn] (EC 1.15.1.1)
Q3T108	Proteasome subunit beta type-4	Q58DM8	Enoyl-CoA hydratase, mitochondrial (mECH) (mECH1) (EC 4.2.1.17) (EC 5.3.3.8) (Enoyl-CoA hydratase 1) (ECHS1) (Short-chain enoyl-CoA hydratase) (SCEH)
Q3T0K2	T-complex protein 1 subunit gamma (TCP-1-gamma) (CCT-gamma)	A0A3Q1MYU9	Sperm acrosome associated 9
Q2T9S0	Tubulin beta-3 chain	P13619	ATP synthase F(0) complex subunit B1, mitochondrial (ATP synthase peripheral stalk-membrane subunit b) (ATP synthase subunit b) (ATPase subunit b)
Q2KJB1	Septin-10	P79136	F-actin-capping protein subunit beta (CapZ beta)
Q1RMJ6	Rho-related GTP-binding protein RhoC	P00125	Cytochrome c1, heme protein, mitochondrial (EC 7.1.1.8) (Complex III subunit 4) (Complex III subunit IV) (Cytochrome b-c1 complex subunit 4) (Ubiquinol-cytochrome-c reductase complex cytochrome c1 subunit) (Cytochrome c-1)
Q148N0	2-oxoglutarate dehydrogenase complex component E1 (E1o) (OGDC-E1) (OGDH-E1) (EC 1.2.4.2) (2-oxoglutarate dehydrogenase, mitochondrial) (Alpha-ketoglutarate dehydrogenase) (Alpha-KGDH-E1) (Thiamine diphosphate (ThDP)-dependent 2-oxoglutarate dehydrogenase)	Q2TA38	Tektin-4
Q0P594	Serine/threonine-protein phosphatase 2A catalytic subunit beta isoform (PP2A-beta) (EC 3.1.3.16)		
Q08D91	Keratin, type II cytoskeletal 75 (Cytokeratin-75) (CK-75) (Keratin-6 hair follicle) (Keratin-75) (K75) (Type II keratin-K6hf) (Type-II keratin Kb18)		
Q02827	NADH dehydrogenase [ubiquinone] 1 subunit C2 (Complex I-B14.5b) (CI-B14.5b) (NADH-ubiquinone oxidoreductase subunit B14.5b)		
P68399	Casein kinase II subunit alpha (CK II alpha) (EC 2.7.11.1)		
O46415	Ferritin light chain (Ferritin L subunit)		
A7YWG4	Gamma-glutamyl hydrolase (EC 3.4.19.9) (Conjugase) (GH)		

	(Gamma-Glu-X carboxypeptidase)
Q3SYR8	Immunoglobulin J chain (Joining chain of multimeric IgA and IgM)
Q17QF5	Protein kinase cAMP-dependent type I regulatory subunit beta (Protein kinase, cAMP-dependent, regulatory, type I, beta)
G5E622	ADAM metallopeptidase domain 20
G3MZX2	Proline rich 30
G3MZ01	Profilin
G3MY97	Golgi associated RAB2 interactor protein-like Rab2B-binding domain-containing protein
F6RFP6	Basigin
F6QJG7	Zona pellucida binding protein 2
F1MYG0	Ornithine aminotransferase (EC 2.6.1.13)
F1MVL2	Acyl-CoA dehydrogenase short chain
F1MPF5	Sperm acrosome developmental regulator
F1MMY0	SSD domain-containing protein
E1BPV0	Armadillo repeat containing 3
E1BPJ2	Coiled-coil domain containing 81
E1BIE7	PGAM family member 5, mitochondrial serine/threonine protein phosphatase
E1BHQ2	Selenoprotein V
E1B993	Ankyrin repeat and EF-hand domain containing 1
A6QPG6	LANCL2 protein (LanC like 2)
A4IFP2	KRT4 protein (Keratin 4)
A0A452DJ99	C-type natriuretic peptide
A0A452DIB4	NADH dehydrogenase [ubiquinone] 1 alpha subcomplex subunit 6 (Complex I-B14) (NADH-ubiquinone oxidoreductase B14 subunit)
A0A3Q1MZQ1	Cytochrome c oxidase subunit 8 (Cytochrome c oxidase polypeptide VIII)
A0A3Q1MZ77	CutA divalent cation tolerance homolog
A0A3Q1MUU9	T-complex protein 1 subunit eta (TCP-1-eta) (CCT-eta)
A0A3Q1ME94	BAF nuclear assembly factor 1
A0A3Q1MDT8	Glutamine amidotransferase class 1 domain containing 3
A0A3Q1MCL0	Follistatin like 5
A0A3Q1M842	Solute carrier family 2, facilitated glucose transporter member 5 (Fructose transporter) (Glucose transporter type 5, small intestine)
A0A3Q1M2V9	Transmembrane protein 11
A0A3Q1M2K0	Chromosome 17 C4orf45 homolog

A0A3Q1LRC3	Cilia and flagella associated protein 43
A0A3Q1LQT3	NADH-cytochrome b5 reductase (EC 1.6.2.2)
A0A3Q1LFR8	Dynein axonemal heavy chain 17
Q02367	NADH dehydrogenase [ubiquinone] 1 beta subcomplex subunit 6 (Complex I-B17) (CI-B17) (NADH-ubiquinone oxidoreductase B17 subunit)
P82908	Alpha-ketoglutarate dehydrogenase component 4
P79136	F-actin-capping protein subunit beta (CapZ beta)
P33097	Aspartate aminotransferase, cytoplasmic (cAspAT) (EC 2.6.1.1) (EC 2.6.1.3) (Cysteine aminotransferase, cytoplasmic) (Cysteine transaminase, cytoplasmic) (cCAT) (Glutamate oxaloacetate transaminase 1) (Transaminase A)
P32007	ADP/ATP translocase 3 (ADP,ATP carrier protein 3) (ADP,ATP carrier protein, isoform T2) (ANT 2) (Adenine nucleotide translocator 3) (ANT 3) (Solute carrier family 25 member 6) [Cleaved into: ADP/ATP translocase 3, N-terminally processed]
P12234	Solute carrier family 25 member 3 (Phosphate carrier protein, mitochondrial) (PiC) (Phosphate transport protein) (PTP)
P05630	ATP synthase subunit delta, mitochondrial (ATP synthase F1 subunit delta) (F-ATPase delta subunit)
P02754	Beta-lactoglobulin (Beta-LG) (allergen Bos d 5)
A7MBH5	Outer dynein arm-docking complex subunit 3 (Coiled-coil domain-containing protein 151)
A0A3Q1LYJ4	Histone H2A
Q2KJD2	Vesicle-associated membrane protein 3 (VAMP-3) (Synaptobrevin-3)
Q2KJI7	AFG3-like protein 2 (EC 3.4.24.-)
A0A3Q1LXA8	inorganic diphosphatase (EC 3.6.1.1)
G3MY76	Cylicin 1
Q32KZ9	Tektin-1
Q2T9S4	Glycerol-3-phosphate phosphatase (G3PP) (EC 3.1.3.21) (Aspartate-based ubiquitous Mg(2+)-dependent phosphatase) (AUM) (EC 3.1.3.48) (Phosphoglycolate phosphatase)

	(PGP)	
F1MX39	RAB42, member RAS oncogene family	
A0A3Q1M1Z1	Histone H4	
F6S1Q0	Keratin 18	
F6QGE6	Acyl-CoA thioesterase 13	
P42029	NADH dehydrogenase [ubiquinone] 1 alpha subcomplex subunit 8 (Complex I-19kD) (CI-19kD) (Complex I-PGIV) (CI-PGIV) (NADH-ubiquinone oxidoreductase 19 kDa subunit)	
Q2TA43	Actin-related protein T2 (ARP-T2)	
P00423	Cytochrome c oxidase subunit 4 isoform 1, mitochondrial (Cytochrome c oxidase polypeptide IV) (Cytochrome c oxidase subunit IV isoform 1) (COX IV-1)	
F1MNL6	Nucleoporin 210 like	
Q8SPU8	Dehydrogenase/reductase SDR family member 4 (EC 1.1.1.184) (EC 1.1.1.300) (NADPH-dependent carbonyl reductase) (CR) (NADPH-dependent retinol dehydrogenase/reductase) (NDRD) (Peroxisomal short-chain alcohol dehydrogenase) (PSCD) (Short chain dehydrogenase/reductase family 25C member 2) (Protein SDR25C2)	
A0A3Q1MYU9	Sperm acrosome associated 9	
Q9MZ13	Voltage-dependent anion-selective channel protein 3 (VDAC-3) (Outer mitochondrial membrane protein porin 3)	
Q32KZ2	Actin-like protein 7A	

Table S3. Upregulated proteins of spermatozoa during late capacitation.

Normal fertility		Reduced fertility	
**Accession No.**	**Gene Name**	**Accession No.**	**Gene Name**
A0JNK3	Serine protease HTRA2, mitochondrial (EC 3.4.21.108)	A0A3Q1M8K4	Integrin subunit alpha 6
A7YWG4	Gamma-glutamyl hydrolase (EC 3.4.19.9) (Conjugase) (GH) (Gamma-Glu-X carboxypeptidase)	A0A3Q1LF83	RING1 and YY1 binding protein
O46415	Ferritin light chain (Ferritin L subunit)	F1MRI2	Mitochondrial import inner membrane translocase subunit Tim21
P05632	ATP synthase subunit epsilon, mitochondrial (ATPase subunit epsilon) (ATP synthase F1 subunit epsilon)	A0A3Q1MUN0	Sorbitol dehydrogenase (SDH) (XDH) (EC 1.1.1.14) (EC 1.1.1.9) (L-iditol 2-dehydrogenase) (Polyol dehydrogenase) (Xylitol dehydrogenase)
P08166	Adenylate kinase 2, mitochondrial (AK 2) (EC 2.7.4.3) (ATP-AMP transphosphorylase 2) (ATP:AMP phosphotransferase) (Adenylate monophosphate kinase) [Cleaved into: Adenylate kinase 2, mitochondrial, N-terminally processed]	E1BAJ3	Coiled-coil domain containing 40
P08904	Ribonuclease K6 (RNase K6) (EC 3.1.27.-) (K6b) (Ribonuclease K2) (RNase K2)	A0A3Q1MWG7	Malic enzyme
P15497	Apolipoprotein A-I (Apo-AI) (ApoA-I) (Apolipoprotein A1) [Cleaved into: Proapolipoprotein A-I (ProapoA-I); Truncated apolipoprotein A-I]	A0A3Q1LRC3	Cilia and flagella associated protein 43
Q02369	NADH dehydrogenase [ubiquinone] 1 beta subcomplex subunit 9 (Complex I-B22) (CI-B22) (NADH-ubiquinone oxidoreductase B22 subunit)	A0A3Q1MAW8	Meiosis-specific nuclear structural protein 1
Q02827	NADH dehydrogenase [ubiquinone] 1 subunit C2 (Complex I-B14.5b) (CI-B14.5b) (NADH-ubiquinone oxidoreductase subunit B14.5b)	Q02367	NADH dehydrogenase [ubiquinone] 1 beta subcomplex subunit 6 (Complex I-B17) (CI-B17) (NADH-ubiquinone oxidoreductase B17 subunit)
Q0II87	Transcription factor A, mitochondrial (mtTFA)	Q6QTG5	Cytochrome c oxidase subunit 3
Q148H7	Keratin, type II cytoskeletal 79 (Cytokeratin-79) (CK-79) (Keratin-79) (K79) (Type-II keratin Kb38)	A0A3Q1MGZ4	Triokinase/FMN cyclase (EC 4.6.1.15) (Bifunctional ATP-dependent dihydroxyacetone kinase/FAD-AMP lyase (cyclizing))
Q1RMJ6	Rho-related GTP-binding protein RhoC	A0A3Q1NJR8	Antithrombin-III (Serpin C1)
Q2KIM0	Tissue alpha-L-fucosidase (EC 3.2.1.51) (Alpha-L-fucosidase I) (Alpha-L-fucoside fucohydrolase 1) (Alpha-L-fucosidase 1)	Q02827	NADH dehydrogenase [ubiquinone] 1 subunit C2 (Complex I-B14.5b) (CI-B14.5b) (NADH-ubiquinone oxidoreductase subunit B14.5b)
Q32KU4	IQ domain-containing protein F5	E1BFM2	Serine protease 50
Q32LJ7	RIB43A-like with coiled-coils protein 2	A0A3Q1LU44	Dynein axonemal heavy chain 12

Q3SZW1	Testis-specific serine/threonine-protein kinase 1 (TSK-1) (TSK1) (TSSK-1) (Testis-specific kinase 1) (EC 2.7.11.1)	A0A3Q1LW84	Protein phosphatase 2 scaffold subunit Aalpha
Q5XQN5	Keratin, type II cytoskeletal 5 (Cytokeratin-5) (CK-5) (Keratin-5) (K5) (Type-II keratin Kb5)	Q2YDE4	Proteasome subunit alpha type-6
A0A3Q1LJ59	ABC transporter domain-containing protein	F1MEM9	Tetratricopeptide repeat domain 19
A0A3Q1LK47	AFG3 like matrix AAA peptidase subunit 2	F1N594	Radial spoke head 14 homolog
A0A3Q1LMN7	[tau protein] kinase (EC 2.7.11.26)	A0A3Q1MP25	Acyl-CoA dehydrogenase family member 9
A0A3Q1LPJ9	ADAM metallopeptidase domain 20	E1BCN8	Phosphoethanolamine/phosphocholine phosphatase 1
A0A3Q1LRV1	Coiled-coil domain containing 183	Q0II87	Transcription factor A, mitochondrial (mtTFA)
A0A3Q1LTA7	Myeloid leukemia factor 1	A0A3Q1LGN5	electron-transferring-flavoprotein dehydrogenase (EC 1.5.5.1)
A0A3Q1LTJ2	Doublecortin domain containing 2C	P08166	Adenylate kinase 2, mitochondrial (AK 2) (EC 2.7.4.3) (ATP-AMP transphosphorylase 2) (ATP:AMP phosphotransferase) (Adenylate monophosphate kinase) [Cleaved into: Adenylate kinase 2, mitochondrial, N-terminally processed]
A0A3Q1LZ21	Hydroxysteroid dehydrogenase like 2	F1MQJ0	Angiotensin-converting enzyme (ACE) (EC 3.4.15.1) (Dipeptidyl carboxypeptidase I) (Kininase II) (CD antigen CD143) [Cleaved into: Angiotensin-converting enzyme, soluble form]
A0A3Q1M2L7	Testis expressed 46	Q5XQN5	Keratin, type II cytoskeletal 5 (Cytokeratin-5) (CK-5) (Keratin-5) (K5) (Type-II keratin Kb5)
A0A3Q1M3C8	H1.7 linker histone	A0A3Q1M3S4	Septin-7
A0A3Q1M4L0	Hsp70-interacting protein N-terminal domain-containing protein	A0A3Q1ND53	Hydroxyacylglutathione hydrolase
A0A3Q1M5Z8	palmitoyl-protein hydrolase (EC 3.1.2.22)	Q0II40	Dual specificity phosphatase 21 (Similar to Dual specificity protein phosphatase 18 (Low molecular weight dual specificity phosphatase 20))
A0A3Q1M7H7	Aspartyl aminopeptidase (EC 3.4.11.21)	A7MBH5	Outer dynein arm-docking complex subunit 3 (Coiled-coil domain-containing protein 151)
A0A3Q1M8K4	Integrin subunit alpha 6	G3MZX2	Proline rich 30
A0A3Q1M9N4	AU RNA binding methylglutaconyl-CoA hydratase	A0A3Q1LHW9	Coilin
A0A3Q1MEM6	PWWP domain containing 3A, DNA repair factor	G3MZM8	vitamin-K-epoxide reductase (warfarin-sensitive) (EC 1.17.4.4)
A0A3Q1MGZ4	Triokinase/FMN cyclase (EC 4.6.1.15) (Bifunctional ATP-dependent dihydroxyacetone kinase/FAD-AMP lyase (cyclizing))	F1MLE3	Conserved oligomeric Golgi complex subunit 6 (COG complex subunit 6) (Component of oligomeric Golgi complex 6)

A0A3Q1ML10	Armadillo repeat containing 3	A6QQM8	Coiled-coil domain-containing protein 42
A0A3Q1ML84	ATP synthase membrane subunit K, mitochondrial	P68399	Casein kinase II subunit alpha (CK II alpha) (EC 2.7.11.1)
A0A3Q1MUN0	Sorbitol dehydrogenase (SDH) (XDH) (EC 1.1.1.14) (EC 1.1.1.9) (L-iditol 2-dehydrogenase) (Polyol dehydrogenase) (Xylitol dehydrogenase)	F1MBE1	Threonine synthase like 1
A0A3Q1MVR8	Multifunctional fusion protein [Includes: Delta-1-pyrroline-5-carboxylate dehydrogenase (P5C dehydrogenase) (L-glutamate gamma-semialdehyde dehydrogenase); L-glutamate gamma-semialdehyde dehydrogenase (EC 1.2.1.88)]	A0A3Q1MNS9	Theg spermatid protein like
A0A3Q1ND53	Hydroxyacylglutathione hydrolase	A0A3Q1MR22	Desmoplakin
A0A452DIK0	NADH dehydrogenase [ubiquinone] 1 alpha subcomplex subunit 12	Q2HJ86	Tubulin alpha-1D chain (EC 3.6.5.-) [Cleaved into: Detyrosinated tubulin alpha-1D chain]
A0A452DIK3	NADH dehydrogenase [ubiquinone] iron-sulfur protein 8, mitochondrial (Complex I-23kD) (NADH-ubiquinone oxidoreductase 23 kDa subunit)	A0A3Q1MEH5	Coiled-coil domain containing 63
A0A452DIM5	Cytochrome c oxidase subunit (Cytochrome c oxidase polypeptide VIa)	A0A3Q1LVP8	Teneurin transmembrane protein 3
A8YXY4	NADH:ubiquinone oxidoreductase subunit S6 (NDUFS6 protein)	A0A452DIW4	Malate dehydrogenase (EC 1.1.1.37)
E1B7I3	ADAM metallopeptidase domain 20	A0A3Q1LXK7	Cytochrome c oxidase subunit 6C (Cytochrome c oxidase polypeptide VIc)
E1B8W3	Outer dynein arm-docking complex subunit 2 (Armadillo repeat-containing protein 4)	A0A3Q1LFK7	Piercer of microtubule wall 2 protein
E1B9F6	Elongation factor 1-alpha	E1BCN4	AMP-dependent synthetase/ligase domain-containing protein
E1BAJ3	Coiled-coil domain containing 40	Q02369	NADH dehydrogenase [ubiquinone] 1 beta subcomplex subunit 9 (Complex I-B22) (CI-B22) (NADH-ubiquinone oxidoreductase B22 subunit)
E1BFM2	Serine protease 50	E1B7Q2	Adenylate kinase 7
F1MKE7	IF rod domain-containing protein	E1B8R6	RBR-type E3 ubiquitin transferase (EC 2.3.2.31)
F1MP48	Chaperonin containing TCP1 subunit 6B	F1MTN5	Dynein light chain Tctex-type 2
F1MR53	Coiled-coil domain containing 27	A0A3Q1LKJ8	Electron transfer flavoprotein subunit beta
F1N3A8	Chromosome 27 C4orf47 homolog	A0A3Q1M6U5	Protein-serine/threonine kinase (EC 2.7.11.-)
F6RLA2	Testis specific serine kinase 4	A0A3Q1M3M6	D-ribitol-5-phosphate cytidylyltransferase (EC 2.7.7.40) (2-C-methyl-D-erythritol 4-

			phosphate cytidylyltransferase-like protein) (Isoprenoid synthase domain-containing protein)
G3X7W8	Keratin 16	E1B993	Ankyrin repeat and EF-hand domain containing 1
Q32P61	Histone H2A	A0A452DIK7	Tubulin alpha chain
Q3ZCK2	RAS like proto-onco A (V-ral simian leukemia viral oncogene homolog A (Ras related))	F1MY93	Leucine rich repeat containing 34
Q5DPW9	Cystatin E/M	A0A3Q1M7H7	Aspartyl aminopeptidase (EC 3.4.11.21)
Q6QTG5	Cytochrome c oxidase subunit 3	E1B7I3	ADAM metallopeptidase domain 20
A0A3Q1M392	Theg spermatid protein	E1BCP9	Galactosidase beta 1 like
A0A3Q1LR88	F-actin-capping protein subunit beta	F1N5R7	Dynein axonemal heavy chain 7
A0A3Q1LPH5	Glyceraldehyde 3-phosphate dehydrogenase, NAD(P) binding domain-containing protein	A0A3Q1LS67	Enoyl-CoA hydratase 1
A0A0A0MP88	Serpin peptidase inhibitor, clade A (alpha-1 antiproteinase, antitrypsin), member 3	A0A3Q1MRI8	Isocitrate dehydrogenase [NAD] subunit, mitochondrial
A6QQ16	NSL1 component of MIS12 kinetochore complex (NSL1 protein)	A0A3Q1M2V9	Transmembrane protein 11
E1BEJ9	NLR family apoptosis inhibitory protein	Q2T9U2	Outer dense fiber protein 2 (Cenexin) (Outer dense fiber of sperm tails protein 2)
Q5E9B1	L-lactate dehydrogenase B chain (LDH-B) (EC 1.1.1.27)	A0A3Q1LUJ9	Glycerophosphodiester phosphodiesterase 1
F1MQJ0	Angiotensin-converting enzyme (ACE) (EC 3.4.15.1) (Dipeptidyl carboxypeptidase I) (Kininase II) (CD antigen CD143) [Cleaved into: Angiotensin-converting enzyme, soluble form]	F1N450	Regulator of G protein signaling 22
A0A3Q1MBV5	X-prolyl aminopeptidase 3	A0A3S5ZPN7	Coiled-coil domain containing 116
F1MLE3	Conserved oligomeric Golgi complex subunit 6 (COG complex subunit 6) (Component of oligomeric Golgi complex 6)	F1MK41	Short chain dehydrogenase/reductase family 39U member 1
A0A3Q1LFK7	Piercer of microtubule wall 2 protein	G3X6L8	Nipsnap homolog 3A
A0A3Q1MYR8	Keratin 3	F1MMY0	SSD domain-containing protein
A0A3Q1M3M6	D-ribitol-5-phosphate cytidylyltransferase (EC 2.7.7.40) (2-C-methyl-D-erythritol 4-phosphate cytidylyltransferase-like protein) (Isoprenoid synthase domain-containing protein)	G3MWG4	isoleucine--tRNA ligase (EC 6.1.1.5) (Isoleucyl-tRNA synthetase)
Q3T0K2	T-complex protein 1 subunit gamma (TCP-1-gamma) (CCT-gamma)	F1N0E5	T-complex protein 1 subunit delta
F1MVB5	Chromosome 23 C6orf136 homolog	A0A3Q1LRV1	Coiled-coil domain containing 183
Q3T0R7	3-ketoacyl-CoA thiolase, mitochondrial (EC 2.3.1.16) (Acetyl-CoA acetyltransferase) (EC	F1N3A8	Chromosome 27 C4orf47 homolog

	2.3.1.9) (Acetyl-CoA acyltransferase) (Acyl-CoA hydrolase, mitochondrial) (EC 3.1.2.-, EC 3.1.2.1, EC 3.1.2.2) (Beta-ketothiolase) (Mitochondrial 3-oxoacyl-CoA thiolase)		
A0A3Q1MCS5	Peptidase S1 domain-containing protein	G5E531	T-complex protein 1 subunit alpha (CCT-alpha)
A0A140T860	Calcineurin like phosphoesterase domain containing 1	Q2HJ81	Tubulin beta-6 chain
A0A3Q1LV36	Importin 5	A0A3Q1MFE4	MICOS complex subunit MIC60 (Mitofilin)
F6S1Q0	Keratin 18	A0A3Q1LW70	Adenylate kinase 8 (EC 2.7.4.6)
G3X6L8	Nipsnap homolog 3A	A0A452DJA3	Cytochrome b-c1 complex subunit 8 (Complex III subunit 8) (Complex III subunit VIII)
F1MZD5	Dehydrogenase/reductase (SDR family) member 4	A0A3Q1MDV9	Rhophilin associated tail protein 1 like
A7MBH5	Outer dynein arm-docking complex subunit 3 (Coiled-coil domain-containing protein 151)	Q32L77	Cilia- and flagella-associated protein 95
P02754	Beta-lactoglobulin (Beta-LG) (allergen Bos d 5)	A0A3Q1MBQ7	Dynein axonemal heavy chain 5
P05630	ATP synthase subunit delta, mitochondrial (ATP synthase F1 subunit delta) (F-ATPase delta subunit)	Q3SZK3	Growth hormone inducible transmembrane protein
P32007	ADP/ATP translocase 3 (ADP,ATP carrier protein 3) (ADP,ATP carrier protein, isoform T2) (ANT 2) (Adenine nucleotide translocator 3) (ANT 3) (Solute carrier family 25 member 6) [Cleaved into: ADP/ATP translocase 3, N-terminally processed]	A0A3Q1MDH5	Acetyl-CoA acyltransferase 2
P79136	F-actin-capping protein subunit beta (CapZ beta)	A0A3Q1MLU2	Sodium channel protein
P82908	Alpha-ketoglutarate dehydrogenase component 4	F1MZB5	Protein SPMIP9 (Sperm microtubule inner protein 9) (Testis-expressed sequence 37 protein) (Testis-specific conserved protein of 21 kDa)
Q02367	NADH dehydrogenase [ubiquinone] 1 beta subcomplex subunit 6 (Complex I-B17) (CI-B17) (NADH-ubiquinone oxidoreductase B17 subunit)	P81019	Seminal plasma protein BSP-30 kDa (BSP-30K)
Q3SWX2	Acyl-coenzyme A thioesterase 9, mitochondrial (Acyl-CoA thioesterase 9) (EC 3.1.2.-) (Acyl-CoA thioester hydrolase 9)	Q32LC5	CKLF-like MARVEL transmembrane domain containing 2
Q6B856	Tubulin beta-2B chain	E1B9U7	Polypeptide N-acetylgalactosaminyltransferase (EC 2.4.1.-) (Protein-UDP acetylgalactosaminyltransferase)
A0A3Q1LFR8	Dynein axonemal heavy chain 17	Q58DG1	MYG1 exonuclease (EC 3.1.-.-)
A0A3Q1LL55	Peptidase S1 domain-containing protein	A0A3Q1MF13	NAD(P)H-hydrate epimerase (EC 5.1.99.6) (Apolipoprotein A-I-binding protein) (AI-BP)

A0A3Q1LNX0	Histone H2B
A0A3Q1LRC3	Cilia and flagella associated protein 43
A0A3Q1M2V9	Transmembrane protein 11
A0A3Q1MDT8	Glutamine amidotransferase class 1 domain containing 3
A0A3Q1ME94	BAF nuclear assembly factor 1
A0A3Q1MR22	Desmoplakin
A0A3Q1MS55	Serpin family C member 1
A0A3Q1NKS6	oxoglutarate dehydrogenase (succinyl-transferring) (EC 1.2.4.2)
A0A452DIB4	NADH dehydrogenase [ubiquinone] 1 alpha subcomplex subunit 6 (Complex I-B14) (NADH-ubiquinone oxidoreductase B14 subunit)
A0A452DJ99	C-type natriuretic peptide
A4IFP2	KRT4 protein (Keratin 4)
A6QPG6	LANCL2 protein (LanC like 2)
E1B993	Ankyrin repeat and EF-hand domain containing 1
E1BIE7	PGAM family member 5, mitochondrial serine/threonine protein phosphatase
F1MMY0	SSD domain-containing protein
F1MPF5	Sperm acrosome developmental regulator
F1MVL2	Acyl-CoA dehydrogenase short chain
F1MW14	RAN guanine nucleotide release factor

	(NAD(P)HX epimerase)
A0A3Q1LUL6	Carboxymuconolactone decarboxylase-like domain-containing protein
P33097	Aspartate aminotransferase, cytoplasmic (cAspAT) (EC 2.6.1.1) (EC 2.6.1.3) (Cysteine aminotransferase, cytoplasmic) (Cysteine transaminase, cytoplasmic) (cCAT) (Glutamate oxaloacetate transaminase 1) (Transaminase A)
G3N284	Actin related protein T1
A0A3Q1M7E3	Glutathione peroxidase
F1MVL2	Acyl-CoA dehydrogenase short chain
A4FV54	Ras-related protein Rab-8A (EC 3.6.5.2)
A0A3Q1LWC1	NADH dehydrogenase [ubiquinone] iron-sulfur protein 2, mitochondrial (Complex I-49kD) (NADH-ubiquinone oxidoreductase 49 kDa subunit)
A7YWG4	Gamma-glutamyl hydrolase (EC 3.4.19.9) (Conjugase) (GH) (Gamma-Glu-X carboxypeptidase)
P00921	Carbonic anhydrase 2 (EC 4.2.1.1) (Carbonate dehydratase II) (Carbonic anhydrase II) (CA-II) (Cyanamide hydratase CA2) (EC 4.2.1.69)
A0A3Q1MQG7	NADH dehydrogenase [ubiquinone] 1 beta subcomplex subunit 8, mitochondrial (Complex I-ASHI) (NADH-ubiquinone oxidoreductase ASHI subunit)
A0A3Q1M7X9	Acyl-CoA thioesterase 9
A0A3Q1NNI3	Phosphotransferase (EC 2.7.1.-)
Q3ZBU2	CDGSH iron-sulfur domain-containing protein 1 (Cysteine transaminase CISD1) (EC 2.6.1.3) (MitoNEET)
A0A3Q1M1M7	Junction plakoglobin
Q2TBS5	Serine peptidase inhibitor, Kazal type 2 (Acrosin-trypsin inhibitor) (Serine peptidase inhibitor, Kazal type 2 B)
F1MDV8	Septin
Q58DU5	Proteasome subunit alpha type-3
A0A3Q1M4Y2	Cilia and flagella associated protein 57

F1MYG0	Ornithine aminotransferase (EC 2.6.1.13)	A0A3Q1MBY4	Protein kinase cAMP-dependent type I regulatory subunit alpha
F6QJG7	Zona pellucida binding protein 2	A0A3Q1LXS2	Cilia and flagella associated protein 161
F6RFP6	Basigin	A0A3Q1MA90	Transmembrane protein 190
G3MY97	Golgi associated RAB2 interactor protein-like Rab2B-binding domain-containing protein	Q6B856	Tubulin beta-2B chain
G3MYJ1	Tetratricopeptide repeat and ankyrin repeat containing 1	A0A3Q1LKE9	Tafazzin family protein
G3MZ71	Keratin 2	F1N431	Farnesyl pyrophosphate synthase ((2E,6E)-farnesyl diphosphate synthase) (Dimethylallyltranstransferase) (Farnesyl diphosphate synthase) (Geranyltranstransferase)
Q17QF5	Protein kinase cAMP-dependent type I regulatory subunit beta (Protein kinase, cAMP-dependent, regulatory, type I, beta)	A0A3Q1M9Q1	NADP-dependent oxidoreductase domain-containing protein
Q3SYR8	Immunoglobulin J chain (Joining chain of multimeric IgA and IgM)	G5E622	ADAM metallopeptidase domain 20
A0A3Q1M842	Solute carrier family 2, facilitated glucose transporter member 5 (Fructose transporter) (Glucose transporter type 5, small intestine)	A0JNK3	Serine protease HTRA2, mitochondrial (EC 3.4.21.108)
A0A3Q1LK04	Ubiquitin carboxyl-terminal hydrolase (EC 3.4.19.12)	Q0P594	Serine/threonine-protein phosphatase 2A catalytic subunit beta isoform (PP2A-beta) (EC 3.1.3.16)
Q3ZBK6	U6 snRNA-associated Sm-like protein LSm4	A0A3Q1M9N4	AU RNA binding methylglutaconyl-CoA hydratase
F1MDV8	Septin	Q1RMJ6	Rho-related GTP-binding protein RhoC
A0A3Q1LSG3	Casein kinase 2 alpha 1	F1MBU8	Dpy-19 like 2
A0A3Q1LYI6	MICOS complex subunit MIC60 (Mitofilin)	E1BEJ9	NLR family apoptosis inhibitory protein
A0A452DIW4	Malate dehydrogenase (EC 1.1.1.37)	A0A3Q1NI70	SLIT-ROBO Rho GTPase activating protein 3
Q2TBX6	Proteasome subunit beta type-1	P11024	NAD(P) transhydrogenase, mitochondrial (EC 7.1.1.1) (Nicotinamide nucleotide transhydrogenase) (Pyridine nucleotide transhydrogenase)
A0A3Q1MWG7	Malic enzyme	A0A452DIM5	Cytochrome c oxidase subunit (Cytochrome c oxidase polypeptide VIa)
G5E531	T-complex protein 1 subunit alpha (CCT-alpha)	Q148D3	Fumarate hydratase, mitochondrial (EC 4.2.1.2)
A0A3Q1LXF6	Cilia and flagella associated protein 58	P67868	Casein kinase II subunit beta (CK II beta) (Phosvitin)
A0A3Q1LWC1	NADH dehydrogenase [ubiquinone] iron-sulfur protein 2, mitochondrial (Complex I-49kD) (NADH-ubiquinone oxidoreductase 49 kDa subunit)	E1B9R5	Dynein axonemal heavy chain 8
A0A3Q1LKJ8	Electron transfer flavoprotein subunit beta	A0A0A0MP88	Serpin peptidase inhibitor, clade A (alpha-1 antiproteinase,

A6H782	Tektin-3
A0A3Q1MRI8	Isocitrate dehydrogenase [NAD] subunit, mitochondrial
A0A3Q1LM20	Histone H4
E1BGD1	Translocase of outer mitochondrial membrane 34
A0A3Q1MEH5	Coiled-coil domain containing 63
Q0VCA3	Mitochondrial proton/calcium exchanger protein (Electroneutral mitochondrial K(+)/H(+)exchanger) (KHE) (Leucine zipper-EF-hand-containing transmembrane protein 1)
Q2TBS5	Serine peptidase inhibitor, Kazal type 2 (Acrosin-trypsin inhibitor) (Serine peptidase inhibitor, Kazal type 2 B)
A0A3Q1M1Z1	Histone H4
P00442	Superoxide dismutase [Cu-Zn] (EC 1.15.1.1)
Q2YDN4	Tektin-like protein 1 (Coiled-coil domain-containing protein 105)
A0A3Q1LXA8	inorganic diphosphatase (EC 3.6.1.1)
P00514	cAMP-dependent protein kinase type I-alpha regulatory subunit [Cleaved into: cAMP-dependent protein kinase type I-alpha regulatory subunit, N-terminally processed]
A0A3Q1MGG8	IQ motif containing N
F1MLS4	Membrane spanning 4-domains A14
G3MY76	Cylicin 1
E1B7X2	Actin like 11
Q32KZ2	Actin-like protein 7A
Q2TA43	Actin-related protein T2 (ARP-T2)
	antitrypsin), member 3
E1B9I5	Cilia- and flagella-associated protein 276
E1BHZ3	FAM161 centrosomal protein A
A0A3Q1M842	Solute carrier family 2, facilitated glucose transporter member 5 (Fructose transporter) (Glucose transporter type 5, small intestine)
Q32KR2	Acrosomal vesicle protein 1
A0A3Q1MIY8	phosphopyruvate hydratase (EC 4.2.1.11) (2-phospho-D-glycerate hydro-lyase)
A8E4N3	Radial spoke head protein 3 homolog (A-kinase anchor protein RSPH3) (Radial spoke head-like protein 2)
A0A3Q1LV18	T-complex protein 1 subunit beta (CCT-beta)
Q32P67	Piercer of microtubule wall 1 protein (Pierce1) (UPF0691 protein C9orf116 homolog)
G3MY97	Golgi associated RAB2 interactor protein-like Rab2B-binding domain-containing protein
E1BPM9	Dynein axonemal intermediate chain 2
P08904	Ribonuclease K6 (RNase K6) (EC 3.1.27.-) (K6b) (Ribonuclease K2) (RNase K2)
Q29S21	Keratin, type II cytoskeletal 7 (Cytokeratin-7) (CK-7) (Keratin-7) (K7) (Type-II keratin Kb7)
A6QPG6	LANCL2 protein (LanC like 2)
A0A3Q1LK47	AFG3 like matrix AAA peptidase subunit 2
P08760	GTP:AMP phosphotransferase AK3, mitochondrial (EC 2.7.4.10) (Adenylate kinase 3) (AK 3) (Adenylate kinase 3 alpha-like 1)
A0A140T860	Calcineurin like phosphoesterase domain containing 1
A0A3Q1N3G0	ABC transporter domain-containing protein
A0A452DIB4	NADH dehydrogenase [ubiquinone] 1 alpha subcomplex subunit 6 (Complex I-B14) (NADH-ubiquinone oxidoreductase B14 subunit)

Q5E9Y9	Nucleoside diphosphate kinase 7 (NDK 7) (NDP kinase 7) (EC 2.7.4.6)
Q2TBQ6	Heat shock protein beta-9 (HspB9)
A0A3Q1LUP0	Phosphatidylethanolamine binding protein 4
F1MUB8	Spermatosis and centriole associated 1
Q9MZ13	Voltage-dependent anion-selective channel protein 3 (VDAC-3) (Outer mitochondrial membrane protein porin 3)
E1B958	ALMS1 centrosome and basal body associated protein
Q02337	D-beta-hydroxybutyrate dehydrogenase, mitochondrial (EC 1.1.1.30) (3-hydroxybutyrate dehydrogenase) (BDH)
A0A3Q1LP28	Enoyl-CoA delta isomerase 2
F1MI43	Sperm surface protein Sp17 (Sperm autoantigenic protein 17)
A0A3Q1M3K6	Leucine-rich repeat-containing protein 37 N-terminal domain-containing protein
P29392	Spermadhesin-1 (Acidic seminal fluid protein) (ASFP)
P05632	ATP synthase subunit epsilon, mitochondrial (ATPase subunit epsilon) (ATP synthase F1 subunit epsilon)
A0A3Q1LF77	Programmed cell death 6
Q08D91	Keratin, type II cytoskeletal 75 (Cytokeratin-75) (CK-75) (Keratin-6 hair follicle) (Keratin-75) (K75) (Type II keratin-K6hf) (Type-II keratin Kb18)
Q17QF5	Protein kinase cAMP-dependent type I regulatory subunit beta (Protein kinase, cAMP-dependent, regulatory, type I, beta)
A0A3Q1LQK0	Cytochrome b5 domain containing 1
A0A3Q1MS98	Acetyl-coenzyme A synthetase (EC 6.2.1.1)
F1N7G5	Cilia- and flagella-associated protein 53
F1N6A0	Isochorismatase domain containing 2
E1BNS6	Sperm-associated antigen 8
Q8HXG6	NADH dehydrogenase [ubiquinone] 1 alpha subcomplex subunit 11 (Complex I-B14.7) (CI-B14.7) (NADH-ubiquinone oxidoreductase subunit B14.7)
A7E3Q2	Heat shock 70kDa protein 1A (Heat shock protein family A (Hsp70) member 2)
P05630	ATP synthase subunit delta, mitochondrial (ATP synthase F1 subunit delta) (F-ATPase delta subunit)
A0A3Q1LRU0	Phospholipase B-like (EC 3.1.1.-)
A6H768	Galactokinase (EC 2.7.1.6) (Galactose kinase)
Q3T0C6	Sodium/potassium-transporting ATPase subunit beta-3 (Sodium/potassium-dependent ATPase subunit beta-3) (ATPB-3) (CD antigen CD298)
F1MH21	Membrane metalloendopeptidase like 1
P06833	Caltrin (Calcium transport inhibitor) (Peptide YY-2) (Peptide YY2) (Seminalplasmin) (SPLN)
A0A3Q1MLP3	Isocitrate dehydrogenase [NAD] subunit, mitochondrial

A0A3Q1LW52	ALMS1 centrosome and basal body associated protein
A0A3Q1N1J5	Phospholipase B-like (EC 3.1.1.-)
F1MPF5	Sperm acrosome developmental regulator
Q02373	NADH dehydrogenase [ubiquinone] 1 beta subcomplex subunit 10 (Complex I-PDSW) (CI-PDSW) (NADH-ubiquinone oxidoreductase PDSW subunit)
A8SMG2	Sperm-associated microtubule inner protein 10 (Testis-expressed protein 43)
E1B836	Enkurin
Q3ZCI1	Transmembrane protein 14C
O46415	Ferritin light chain (Ferritin L subunit)
Q148N0	2-oxoglutarate dehydrogenase complex component E1 (E1o) (OGDC-E1) (OGDH-E1) (EC 1.2.4.2) (2-oxoglutarate dehydrogenase, mitochondrial) (Alpha-ketoglutarate dehydrogenase) (Alpha-KGDH-E1) (Thiamine diphosphate (ThDP)-dependent 2-oxoglutarate dehydrogenase)
P42029	NADH dehydrogenase [ubiquinone] 1 alpha subcomplex subunit 8 (Complex I-19kD) (CI-19kD) (Complex I-PGIV) (CI-PGIV) (NADH-ubiquinone oxidoreductase 19 kDa subunit)
F1N3R5	Aquaporin 7
P42026	NADH dehydrogenase [ubiquinone] iron-sulfur protein 7, mitochondrial (EC 7.1.1.2) (Complex I-20kD) (CI-20kD) (NADH-ubiquinone oxidoreductase 20 kDa subunit) (PSST subunit)
F1MGC0	Succinate--CoA ligase [ADP-forming] subunit beta, mitochondrial (EC 6.2.1.5) (ATP-specific succinyl-CoA synthetase subunit beta) (A-SCS) (Succinyl-CoA synthetase beta-A chain) (SCS-betaA)
Q3MHW6	Monocarboxylate transporter 1 (MCT 1) (Solute carrier family 16 member 1)
Q2YDN8	Inactive serine/threonine-protein kinase VRK3 (Serine/threonine-protein pseudokinase VRK3) (Vaccinia-related kinase 3)
P82908	Alpha-ketoglutarate dehydrogenase component 4
G3X7W8	Keratin 16
F6QJG7	Zona pellucida binding protein 2

P02754	Beta-lactoglobulin (Beta-LG) (allergen Bos d 5)
A0A3Q1MVR8	Multifunctional fusion protein [Includes: Delta-1-pyrroline-5-carboxylate dehydrogenase (P5C dehydrogenase) (L-glutamate gamma-semialdehyde dehydrogenase); L-glutamate gamma-semialdehyde dehydrogenase (EC 1.2.1.88)]
A0A0N4STN0	ATP synthase subunit
A0A3Q1LL55	Peptidase S1 domain-containing protein
A0A3Q1LYI6	MICOS complex subunit MIC60 (Mitofilin)
A6H782	Tektin-3
A0A3Q1MS55	Serpin family C member 1
E1BLC8	Eukaryotic translation initiation factor 4 gamma 3
A0A3Q1LSG3	Casein kinase 2 alpha 1
A6QQ16	NSL1 component of MIS12 kinetochore complex (NSL1 protein)
Q3T145	Malate dehydrogenase, cytoplasmic (EC 1.1.1.37) (Aromatic alpha-keto acid reductase) (KAR) (EC 1.1.1.96) (Cytosolic malate dehydrogenase)
P00442	Superoxide dismutase [Cu-Zn] (EC 1.15.1.1)
Q2TA43	Actin-related protein T2 (ARP-T2)
Q2HJD7	3-hydroxyisobutyrate dehydrogenase, mitochondrial (HIBADH) (EC 1.1.1.31)
P63026	Vesicle-associated membrane protein 2 (VAMP-2) (Synaptobrevin-2)
A0JNM2	Thioredoxin
F1MNQ4	Superoxide dismutase [Cu-Zn] (EC 1.15.1.1)
F1MI43	Sperm surface protein Sp17 (Sperm autoantigenic protein 17)
F1N369	Zona pellucida binding protein
F1MTN9	NME/NM23 family member 8
A0A3Q1LX99	Phosphatidylethanolamine-binding protein 1
P34933	Heat shock-related 70 kDa protein 2 (Heat shock 70 kDa protein 3) (HSP70.3)
A0A3Q1LUP0	Phosphatidylethanolamine binding protein 4
G5E5K9	Mitochondria-eating protein (Spermatogenesis-associated protein 18)
F6S1Q0	Keratin 18
F1MEI0	Fibrous sheath CABYR binding protein
Q32L61	Calcium binding tyrosine

		phosphorylation regulated (Calcium binding tyrosine-(Y)-phosphorylation regulated)
	F1MGY9	Aspartylglucosaminidase
	Q0VCU3	Cathepsin F
	F1MZM9	Hyaluronidase (EC 3.2.1.35)
	Q9BGI1	Peroxiredoxin-5, mitochondrial (EC 1.11.1.24) (Peroxiredoxin V) (Prx-V) (Thioredoxin peroxidase) (Thioredoxin-dependent peroxiredoxin 5)
	Q17QK3	Carboxypeptidase Q (EC 3.4.17.-) (Plasma glutamate carboxypeptidase)
	F1MJS8	A-kinase anchoring protein 3
	Q3SZT4	Testis-expressed protein 29
	F1MFB3	Gem-associated protein 6
	F1MWG1	oxoglutarate dehydrogenase (succinyl-transferring) (EC 1.2.4.2)
	G3N0Z0	IQ motif containing F6
	A1A4J1	ATP-dependent 6-phosphofructokinase, liver type (ATP-PFK) (PFK-L) (EC 2.7.1.11) (6-phosphofructokinase type B) (Phosphofructo-1-kinase isozyme B) (PFK-B) (Phosphohexokinase)
	Q2T9S4	Glycerol-3-phosphate phosphatase (G3PP) (EC 3.1.3.21) (Aspartate-based ubiquitous Mg(2+)-dependent phosphatase) (AUM) (EC 3.1.3.48) (Phosphoglycolate phosphatase) (PGP)

Table S4. Identified transcripts associated mitochondrial and cytoplasmic ribosomal subunits in spermatozoa.

Mitochondrial ribosomal subunits			Cytoplasmic ribosomal subunits		
Enssenble	Gene ID	Read count	Enssenble	Gene ID	Read count
NM_001076307_1	MRRF	28.0	NM_174788	RPLP2	16.8
NM_001035332	MRPS9	19.3	NM_001025340	RPLP1	145.2
NM_001034343	MRPS7	17.2	NM_001012682	RPLP0	17.2
NM_001040584	MRPS6	16.6	NM_001024469	RPL9	31.8
NM_001046123	MRPS5	3.2	NM_001034625	RPL8	20.9
NM_001075619	MRPS35	4.7	NM_001206301	RPL7L1	28.5
NM_001037605	MRPS33	8.8	NM_001040520	RPL7A	31.0
NM_001076015	MRPS31	3.7	NM_001014928	RPL7	23.3
NM_001079595	MRPS30	8.6	NM_001031756	RPL6	76.3
NM_001046401	MRPS28	19.7	NM_001035306	RPL5	14.9
NM_001038108	MRPS27	18.9	NM_001014894	RPL4	17.3
NM_001034514	MRPS26	20.3	NM_001035501	RPL3L	5.7
NM_001046601	MRPS24	9.4	NM_001105455	RPL39	64.0
NM_001046192	MRPS23	6.7	NM_001078132	RPL37	75.3
NM_001046319	MRPS22	58.3	NM_001079508	RPL36AL	38.9
NM_001113307	MRPS21	19.7	NM_001034316	RPL36A	30.1
NM_001046508	MRPS18C	30.4	NM_001078139	RPL36	21.6
NM_001038524	MRPS18B	2.5	NM_001034495	RPL35	43.5
NM_001075474	MRPS16	9.0	NM_001206221	RPL34	10.0
NM_001003902	MRPS14	10.1	NM_001034434	RPL30	8.8
NM_001035078	MRPS11	6.0	NM_174715	RPL3	5.7
NM_001034787	MRPL57	45.8	NM_001034051	RPL27	15.3
NM_001303495	MRPL55	18.0	NM_001015512	RPL26	63.1
NM_001035105	MRPL54	21.2	NM_001045958	RPL23A	123.4
NM_001045926	MRPL53	8.7	NM_001035014	RPL23	31.6
NM_001110180	MRPL52	23.3	NM_001098131	RPL22L1	8.7
NM_001101088	MRPL51	9.7	NM_001191412	RPL21	5.1
NM_001046180	MRPL50	20.8	NM_001033619	RPL18A	10.3
NM_001014871	MRPL49	29.1	NM_001015556	RPL18	10.7
NM_001046563	MRPL48	2.9	NM_001034459	RPL17	41.4
NM_001076901	MRPL47	5.5	NM_001077866	RPL15	28.8
NM_001034712	MRPL46	10.5	NM_001076998	RPL13A	19.3
NM_001035100	MRPL45	7.1	NM_001015543	RPL13	76.0
NM_001046321	MRPL44	17.2	NM_205797	RPL12	34.2
NM_174563	MRPL43	5.8	NM_001075581	RPL11	54.7
NM_001099365	MRPL41	25.0	NM_001205401	RPL10L	72.5
NM_001037450	MRPL4	14.7	NM_001015647	RPL10A	30.9
NM_001035489	MRPL38	3.4	NM_174760	RPL10	38.0
NM_001083400	MRPL37	9.8	NM_001101152	RPS9	40.1
NM_001192391	MRPL35	19.8	NM_001037472	RPS6KL1	1.4
NM_001110187	MRPL34	4.9	NM_001193022	RPS6KC1	48.8
NM_001113308	MRPL33	87.1	NM_001192023	RPS6KA5	15.7
NM_001038679	MRPL32	15.3	NM_001098937	RPS6KA3	1.4
NM_001045996	MRPL28	13.8	NM_001192478	RPS6KA2	1.6
NM_001034576	MRPL24	10.3	NM_001083722	RPS6KA1	2.0
NM_001077884_1	MRPL23	11.9	NM_001015548	RPS6	62.2
NM_001038506	MRPL20	4.5	NM_001015548_1	RPS6	46.1
NM_001046068	MRPL19	19.7	NM_001015531	RPS5	16.8
NM_001034274	MRPL17	7.1	NM_001082422	RPS4Y1	40.7
NM_001075445	MRPL15	13.2	NM_001035445	RPS4X	78.8
NM_001076337	MRPL14	32.8	NM_001034038	RPS3A	12.7
NM_001034805	MRPL13	29.4	NM_174778	RPS27A	17.2
NM_001192395	MRPL10	21.1	NR_024614	RPS27	12.1
NM_001101844	MRPL1	12.1	NM_001015561	RPS26	33.9
			NM_001025315	RPS25	50.5

	NM_001025339	RPS24	12.9
	NM_001034690	RPS23	11.1
	NM_001034438	RPS20	47.1
	NM_001033613	RPS2	32.2
	NM_001037467	RPS19	42.5
	NM_001033614	RPS18	33.3
	NM_001099210	RPS17	7.9
	NM_001033624	RPS16	18.5
	NM_001037443	RPS15A	42.6
	NM_001025342	RPS13	7.2
	NM_001014387	RPS12	32.9
	NM_001034716	RPS10	35.8

Figure S1

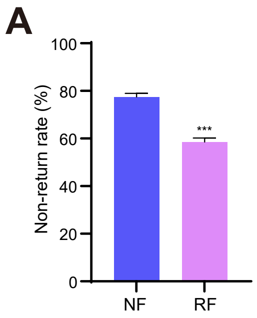
A
Non-return rate (%)
100
80
60
40
20
0
***
NF
RF

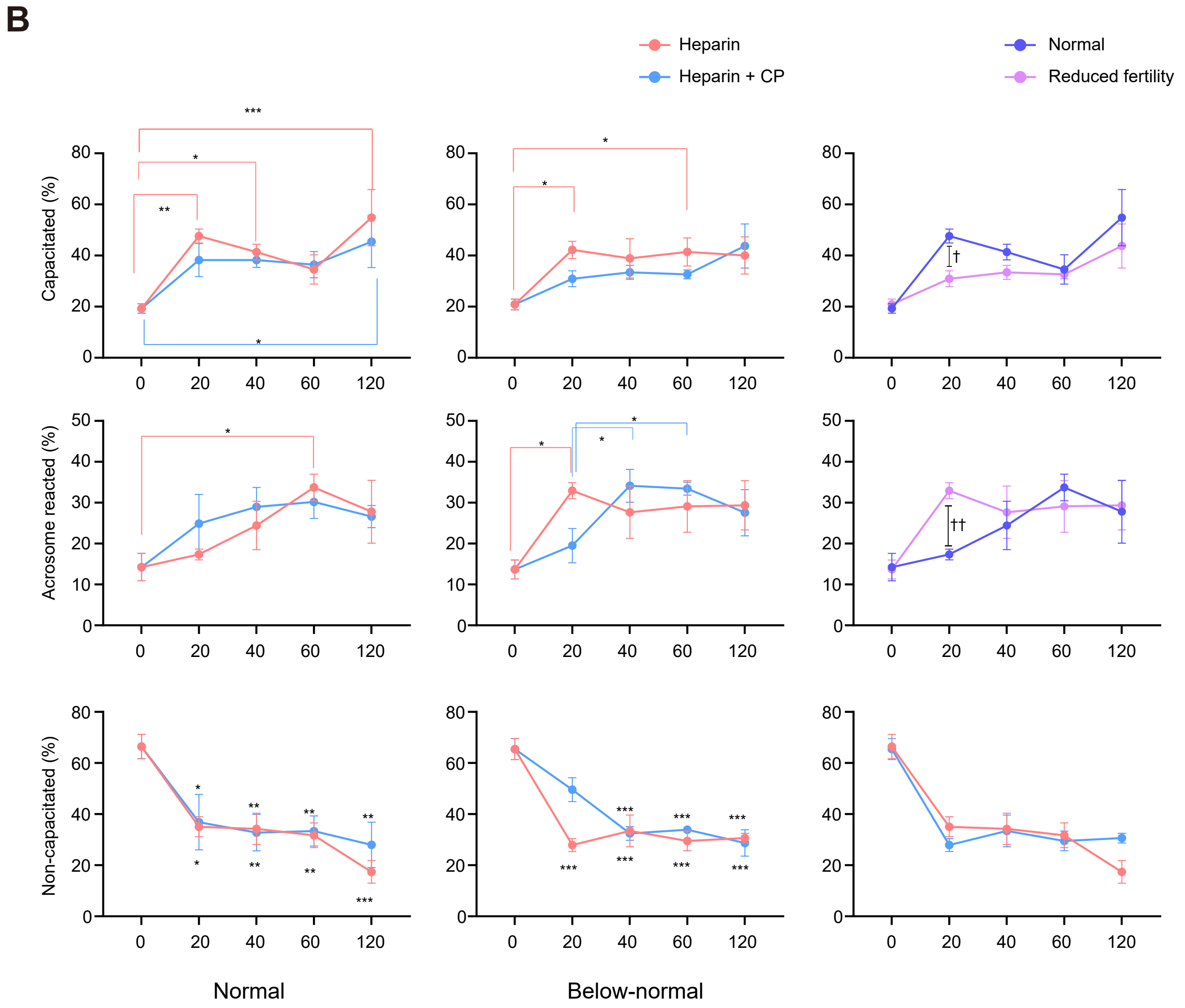
B
Heparin
Heparin + CP
Normal
Reduced fertility
Capacitated (%)
Acrosome reacted (%)
Non-capacitated (%)
0
20
40
60
120
†
††
Normal
Below-normal

Figure S2

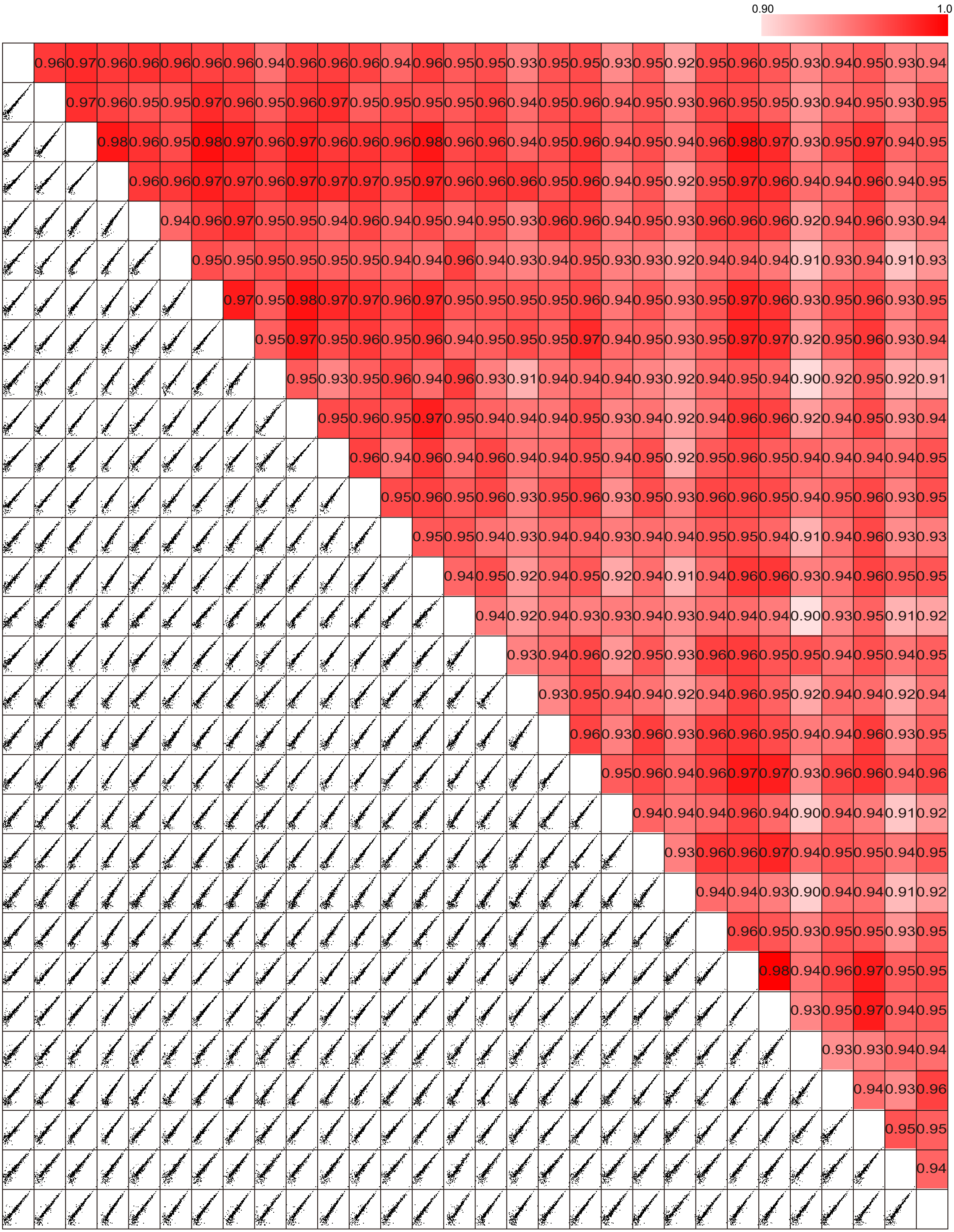
0.90
1.0
0.96 0.97 0.96 0.96 0.96 0.96 0.96 0.94 0.96 0.96 0.96 0.94 0.96 0.95 0.95 0.93 0.95 0.95 0.93 0.95 0.92 0.95 0.96 0.95 0.93 0.94 0.95 0.93 0.94
0.97 0.96 0.95 0.95 0.97 0.96 0.95 0.96 0.97 0.95 0.95 0.95 0.95 0.96 0.94 0.95 0.96 0.94 0.95 0.93 0.96 0.95 0.95 0.93 0.94 0.95 0.93 0.95
0.98 0.96 0.95 0.98 0.97 0.96 0.97 0.96 0.96 0.96 0.98 0.96 0.96 0.94 0.95 0.96 0.94 0.95 0.94 0.96 0.98 0.97 0.93 0.95 0.97 0.94 0.95
0.96 0.96 0.97 0.97 0.96 0.97 0.97 0.97 0.95 0.97 0.96 0.96 0.96 0.95 0.96 0.94 0.95 0.92 0.95 0.97 0.96 0.94 0.94 0.96 0.94 0.95
0.94 0.96 0.97 0.95 0.95 0.94 0.96 0.94 0.95 0.94 0.95 0.93 0.96 0.96 0.94 0.95 0.93 0.96 0.96 0.96 0.92 0.94 0.96 0.93 0.94
0.95 0.95 0.95 0.95 0.95 0.95 0.94 0.94 0.96 0.94 0.93 0.94 0.95 0.93 0.93 0.92 0.94 0.94 0.94 0.91 0.93 0.94 0.91 0.93
0.97 0.95 0.98 0.97 0.97 0.96 0.97 0.95 0.95 0.95 0.95 0.96 0.94 0.95 0.93 0.95 0.97 0.96 0.93 0.95 0.96 0.93 0.95
0.95 0.97 0.95 0.96 0.95 0.96 0.94 0.95 0.95 0.95 0.97 0.94 0.95 0.93 0.95 0.97 0.97 0.92 0.95 0.96 0.93 0.94
0.95 0.93 0.95 0.96 0.94 0.96 0.93 0.91 0.94 0.94 0.94 0.93 0.92 0.94 0.95 0.94 0.90 0.92 0.95 0.92 0.91
0.95 0.96 0.95 0.97 0.95 0.94 0.94 0.94 0.95 0.93 0.94 0.92 0.94 0.96 0.96 0.92 0.94 0.95 0.93 0.94
0.96 0.94 0.96 0.94 0.96 0.94 0.94 0.95 0.94 0.95 0.92 0.95 0.96 0.95 0.94 0.94 0.94 0.94 0.95
0.95 0.96 0.95 0.96 0.93 0.95 0.96 0.93 0.95 0.93 0.96 0.96 0.95 0.94 0.95 0.96 0.93 0.95
0.95 0.95 0.94 0.93 0.94 0.94 0.93 0.94 0.94 0.95 0.95 0.94 0.91 0.94 0.96 0.93 0.93
0.94 0.95 0.92 0.94 0.95 0.92 0.94 0.91 0.94 0.96 0.96 0.93 0.94 0.96 0.95 0.95
0.94 0.92 0.94 0.93 0.93 0.94 0.93 0.94 0.94 0.94 0.90 0.93 0.95 0.91 0.92
0.93 0.94 0.96 0.92 0.95 0.93 0.96 0.96 0.95 0.95 0.94 0.95 0.94 0.95
0.93 0.95 0.94 0.94 0.92 0.94 0.96 0.95 0.92 0.94 0.94 0.92 0.94
0.96 0.93 0.96 0.93 0.96 0.96 0.95 0.94 0.94 0.96 0.93 0.95
0.95 0.96 0.94 0.96 0.97 0.97 0.93 0.96 0.96 0.94 0.96
0.94 0.94 0.94 0.96 0.94 0.90 0.94 0.94 0.91 0.92
0.93 0.96 0.96 0.97 0.94 0.95 0.95 0.94 0.95
0.94 0.94 0.93 0.90 0.94 0.94 0.91 0.92
0.96 0.95 0.93 0.95 0.95 0.93 0.95
0.98 0.94 0.96 0.97 0.95 0.95
0.93 0.95 0.97 0.94 0.95
0.93 0.93 0.94 0.94
0.94 0.93 0.96
0.95 0.95
0.94

Figure S3

A

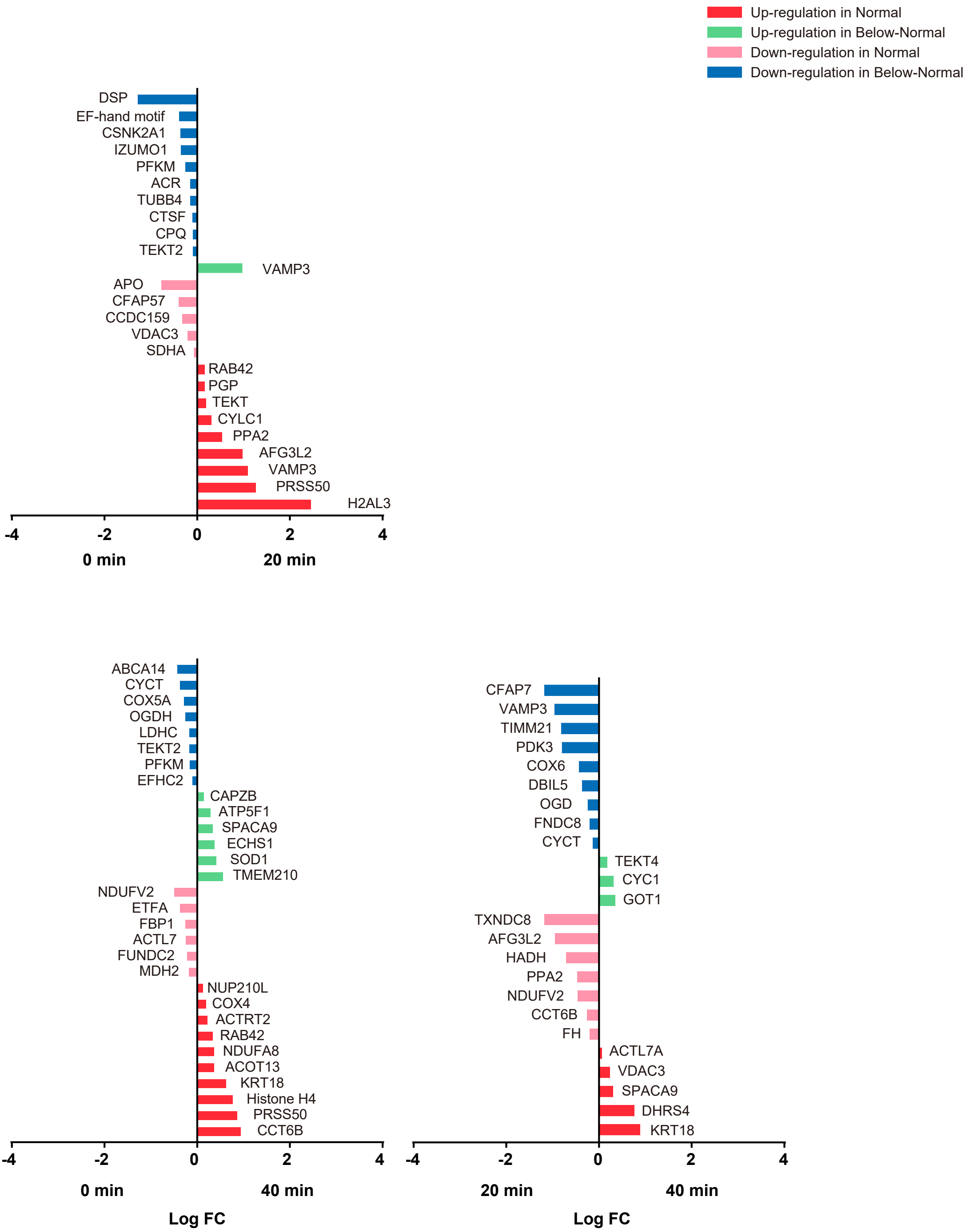

Up-regulation in Normal
Up-regulation in Below-Normal
Down-regulation in Normal
Down-regulation in Below-Normal
DSP
EF-hand motif
CSNK2A1
IZUMO1
PFKM
ACR
TUBB4
CTSF
CPQ
TEKT2
VAMP3
APO
CFAP57
CCDC159
VDAC3
SDHA
RAB42
PGP
TEKT
CYLC1
PPA2
AFG3L2
VAMP3
PRSS50
H2AL3
-4 -2 0 2 4
0 min
20 min
ABCA14
CYCT
COX5A
OGDH
LDHC
TEKT2
PFKM
EFHC2
CAPZB
ATP5F1
SPACA9
ECHS1
SOD1
TMEM210
NDUFV2
ETFA
FBP1
ACTL7
FUNDC2
MDH2
NUP210L
COX4
ACTRT2
RAB42
NDUFA8
ACOT13
KRT18
Histone H4
PRSS50
CCT6B
-4 -2 0 2 4
0 min
40 min
Log FC
CFAP7
VAMP3
TIMM21
PDK3
COX6
DBIL5
OGD
FNDC8
CYCT
TEKT4
CYC1
GOT1
TXNDC8
AFG3L2
HADH
PPA2
NDUFV2
CCT6B
FH
ACTL7A
VDAC3
SPACA9
DHRS4
KRT18
-4 -2 0 2 4
20 min
40 min
Log FC

Figure S3

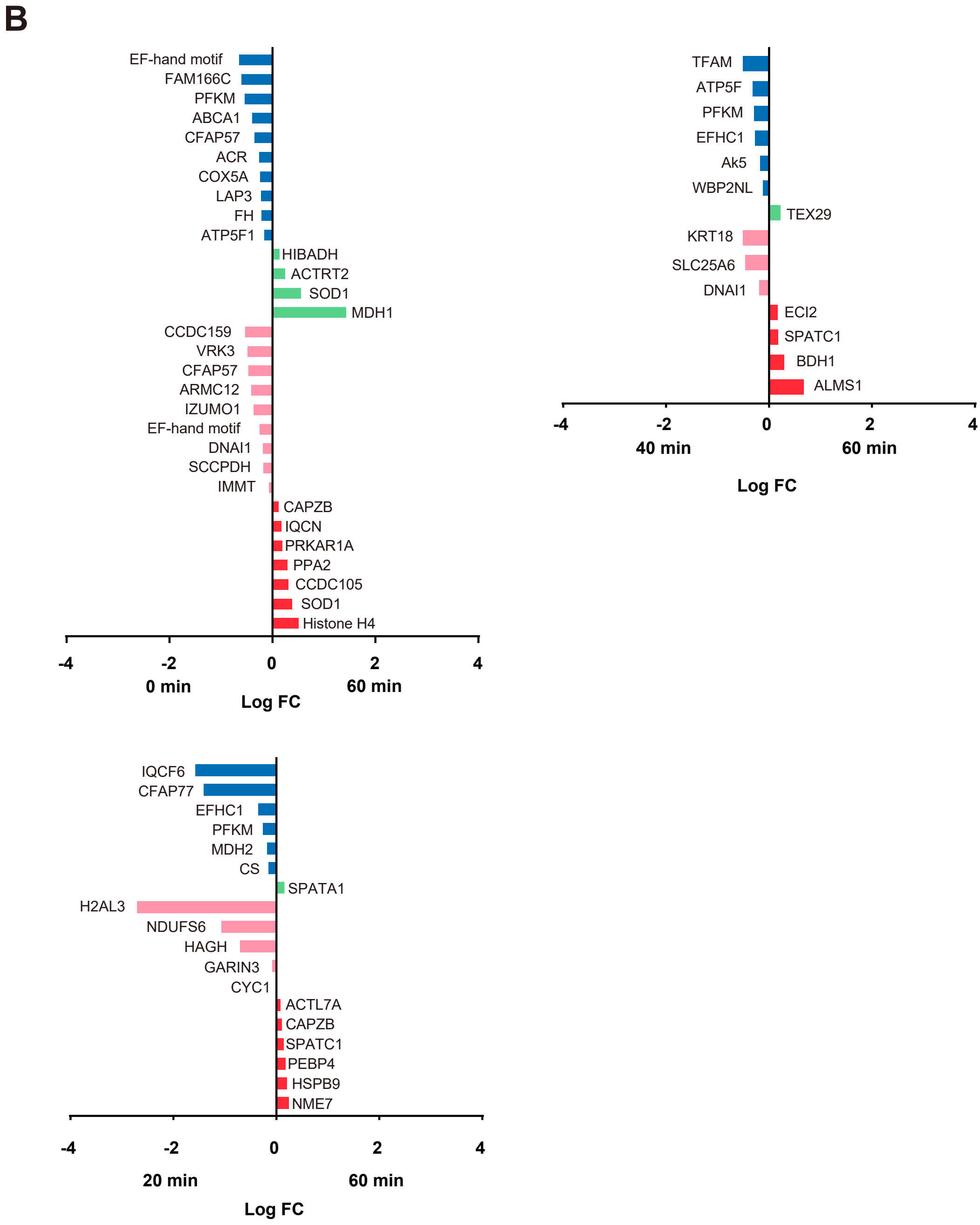
B
EF-hand motif
FAM166C
PFKM
ABCA1
CFAP57
ACR
COX5A
LAP3
FH
ATP5F1
HIBADH
ACTRT2
SOD1
MDH1
CCDC159
VRK3
CFAP57
ARMC12
IZUMO1
EF-hand motif
DNAI1
SCCPDH
IMMT
CAPZB
IQCN
PRKAR1A
PPA2
CCDC105
SOD1
Histone H4
-4 -2 0 2 4
0 min
60 min
Log FC
TFAM
ATP5F
PFKM
EFHC1
Ak5
WBP2NL
TEX29
KRT18
SLC25A6
DNAI1
ECI2
SPATC1
BDH1
ALMS1
-4 -2 0 2 4
40 min
60 min
Log FC
IQCF6
CFAP77
EFHC1
PFKM
MDH2
CS
SPATA1
H2AL3
NDUFS6
HAGH
GARIN3
CYC1
ACTL7A
CAPZB
SPATC1
PEBP4
HSPB9
NME7
-4 -2 0 2 4
20 min
60 min
Log FC

Figure S3

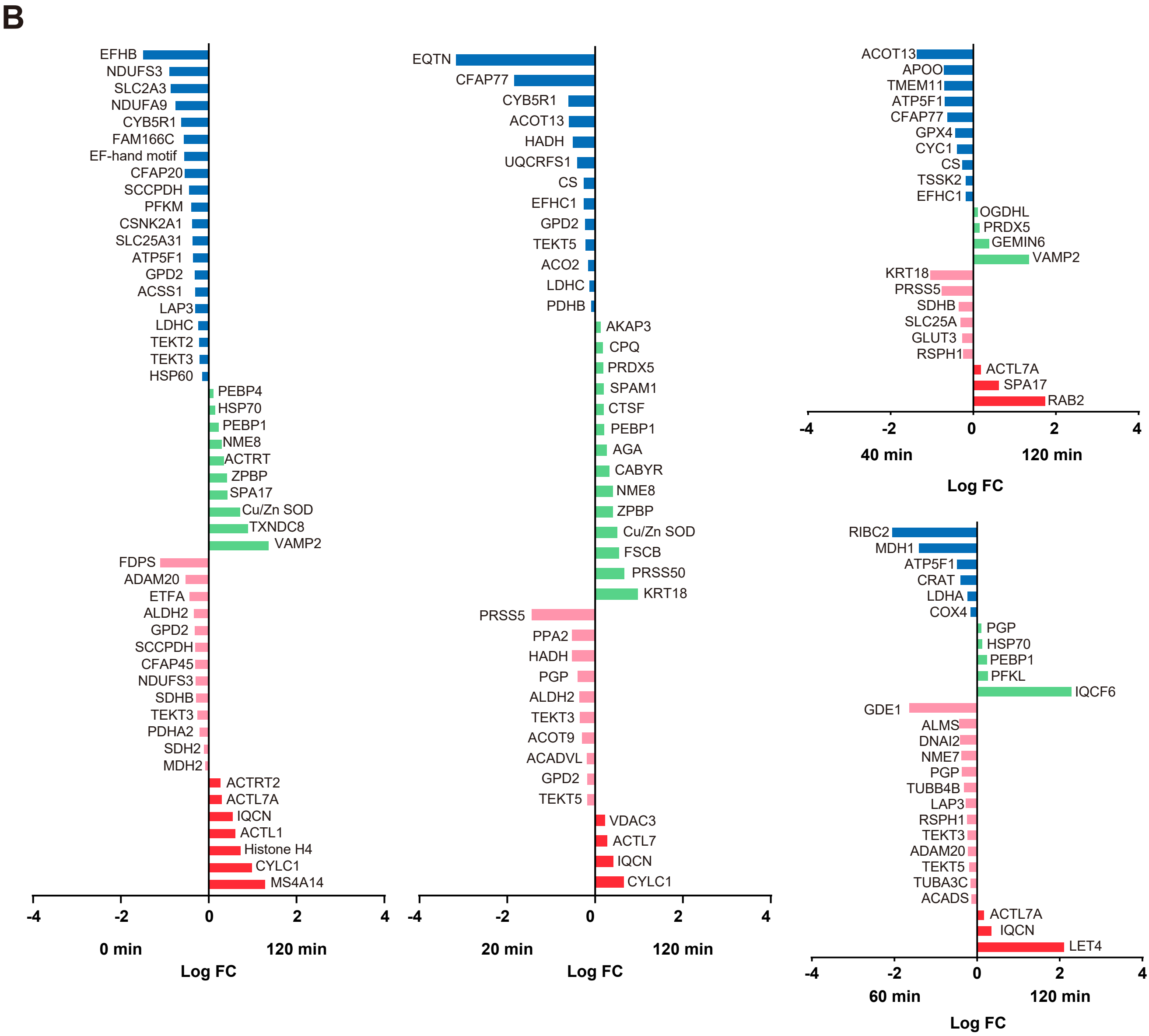
B
EFHB
NDUFS3
SLC2A3
NDUFA9
CYB5R1
FAM166C
EF-hand motif
CFAP20
SCCPDH
PFKM
CSNK2A1
SLC25A31
ATP5F1
GPD2
ACSS1
LAP3
LDHC
TEKT2
TEKT3
HSP60
PEBP4
HSP70
PEBP1
NME8
ACTRT
ZPBP
SPA17
Cu/Zn SOD
TXNDC8
VAMP2
FDPS
ADAM20
ETFA
ALDH2
GPD2
SCCPDH
CFAP45
NDUFS3
SDHB
TEKT3
PDHA2
SDH2
MDH2
ACTRT2
ACTL7A
IQCN
ACTL1
Histone H4
CYLC1
MS4A14
-4 -2 0 2 4
0 min
120 min
Log FC
EQTN
CFAP77
CYB5R1
ACOT13
HADH
UQCRFS1
CS
EFHC1
GPD2
TEKT5
ACO2
LDHC
PDHB
AKAP3
CPQ
PRDX5
SPAM1
CTSF
PEBP1
AGA
CABYR
NME8
ZPBP
Cu/Zn SOD
FSCB
PRSS50
KRT18
PRSS5
PPA2
HADH
PGP
ALDH2
TEKT3
ACOT9
ACADVL
GPD2
TEKT5
VDAC3
ACTL7
IQCN
CYLC1
-4 -2 0 2 4
20 min
120 min
Log FC
ACOT13
APOO
TMEM11
ATP5F1
CFAP77
GPX4
CYC1
CS
TSSK2
EFHC1
OGDHL
PRDX5
GEMIN6
VAMP2
KRT18
PRSS5
SDHB
SLC25A
GLUT3
RSPH1
ACTL7A
SPA17
RAB2
-4 -2 0 2 4
40 min
120 min
Log FC
RIBC2
MDH1
ATP5F1
CRAT
LDHA
COX4
PGP
HSP70
PEBP1
PFKL
IQCF6
GDE1
ALMS
DNAI2
NME7
PGP
TUBB4B
LAP3
RSPH1
TEKT3
ADAM20
TEKT5
TUBA3C
ACADS
ACTL7A
IQCN
LET4
-4 -2 0 2 4
60 min
120 min
Log FC

Figure S4

2h incubation_acrosome intacted

2h incubation_acrosome reacted

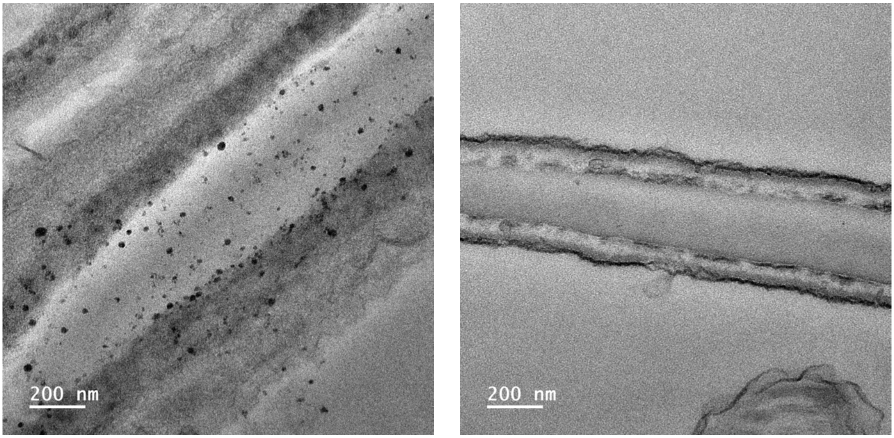

Figure S5

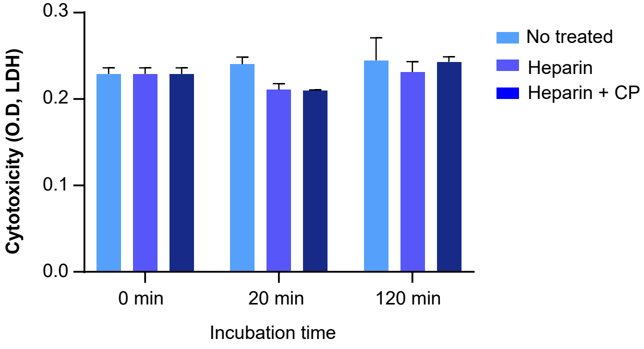

0.3
0.2
0.1
0.0
Cytotoxicity (O.D, LDH)
0 min
20 min
120 min
Incubation time
No treated
Heparin
Heparin + CP
